# Specific Lipid Studies in Complex Membranes by Solid‐State NMR Spectroscopy

**DOI:** 10.1002/chem.202202472

**Published:** 2022-10-25

**Authors:** Roy A. M. van Beekveld, Maik G. N. Derks, Raj Kumar, Leanna Smid, Thorben Maass, João Medeiros‐Silva, Eefjan Breukink, Markus Weingarth

**Affiliations:** ^1^ NMR Spectroscopy Department of Chemistry Faculty of Science Utrecht University Padualaan 8 3584 CH Utrecht The Netherlands; ^2^ Membrane Biochemistry and Biophysics Department of Chemistry Faculty of Science Utrecht University Padualaan 8 3584 CH Utrecht The Netherlands; ^3^ Present address: Department of Chemistry Massachusetts Institute of Technology 170 Albany Street Cambridge MA 02139 USA

**Keywords:** antibiotics, cinnamycin, lipids, membranes, solid state NMR

## Abstract

Specific interactions with phospholipids are often critical for the function of proteins or drugs, but studying these interactions at high resolution remains difficult, especially in complex membranes that mimic biological conditions. In principle, molecular interactions with phospholipids could be directly probed by solid‐state NMR (ssNMR). However, due to the challenge to detect specific lipids in mixed liposomes and limited spectral sensitivity, ssNMR studies of specific lipids in complex membranes are scarce. Here, by using purified biological ^13^C,^15^N‐labeled phospholipids, we show that we can selectively detect traces of specific lipids in complex membranes. In combination with ^1^H‐detected ssNMR, we show that our approach provides unprecedented high‐resolution insights into the mechanisms of drugs that target specific lipids. This broadly applicable approach opens new opportunities for the molecular characterization of specific lipid interactions with proteins or drugs in complex fluid membranes.

## Graphical abstract

**Specific lipid interaction in complex membranes**: Specific interactions with lipids are critical for proteins and drugs, however, direct structural measurements of interactions with lipids remain difficult, especially in membranes that mimic biological conditions. Here, a broadly applicable solid‐state NMR approach is presented that enables to directly measure specific interactions with lipids such as cardiolipin in complex fluid membranes.

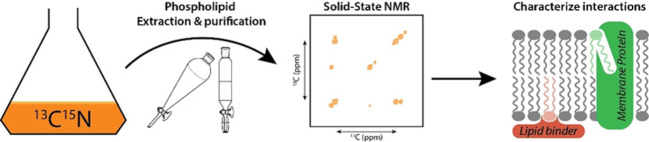

## Introduction

Specific interactions with phospholipids are essential for the function of living cells, for example, by modulating membrane proteins^[1]^ or by shaping membrane properties.^[2]^ Furthermore, an increasingly large number of drugs target specific phospholipids.^[3]^ However, the study of specific interactions with phospholipids at atomic resolution remains a challenge, particularly in complex fluid membranes that mimic biological conditions. This is due to the general difficulty to work in membranes, and due to the dynamic nature of small lipid molecules that evade most structural biology methods.

Solid‐state NMR (ssNMR) could be an ideal method to investigate specific phospholipids^[4]^ in complex membranes, but such studies are scarce. This is due to the challenge to distinguish specific phospholipids in mixed membranes and due to insufficient ssNMR signal sensitivity. These problems could be overcome with ^13^C,^15^N‐labeled phospholipids that could be selectively detected with high sensitivity in mixed membranes. However, uniformly ^13^C,^15^N‐labeled phospholipids are not commercially available, and their purification has hitherto not been reported, severely curtailing the scope of ssNMR studies of phospholipids. What is more, next to the problem of isotope‐labeling, synthetic phospholipids sometimes lack critical properties of biological phospholipids, such as headgroup stereochemistry and lipid‐tail features. Synthetic phospholipids usually contain defined uniform lipid tails, while biological lipids have complex tails.^[5]^

Here, we report the purification of uniformly ^13^C,^15^N‐labeled phospholipids (henceforth called ^*13*^ *C,^15^N‐lipids*) from cells, including lipids of high biological importance. We show that we can detect traces of ^13^C,^15^N‐lipids in mixed membranes. Furthermore, we show that, in combination with modern ^1^H‐detected ssNMR,^[6]^ our approach provides unprecedented molecular insights into the mechanisms of drugs that target specific lipids in complex membranes.

**Figure 1:**
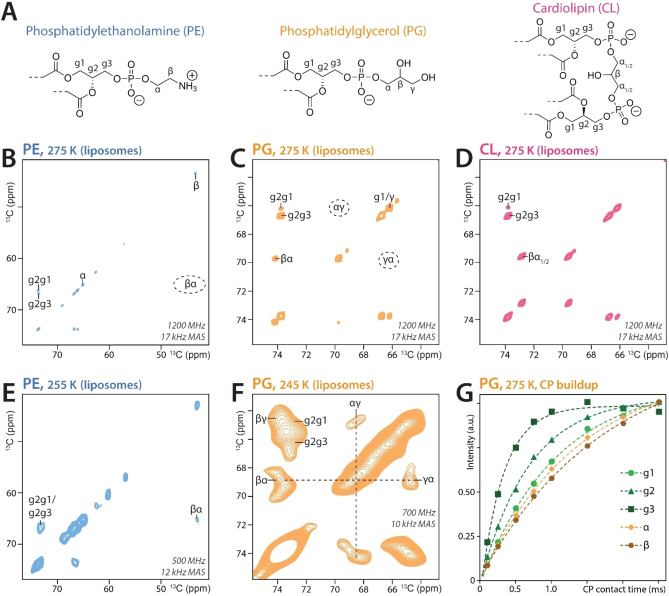
ssNMR characterization of phospholipid headgroups. A) Chemical structures of PE, PG, and cardiolipin lipid‐headgroups. B–D) Zoom into the lipid headgroup region of 2D CC PARISxy ssNMR spectra (200 ms CC magnetization transfer time) acquired with 5 mol % of B) ^13^C,^15^N‐PE, C) ^13^C‐PG, and D) ^13^C‐cardiolipin in DOPC liposomes at 1200 MHz magnetic field and a sample temperature of 275 K. E) 2D CC (150 ms CC transfer time) of ^13^C,^15^N‐PE in DOPC liposomes acquired at 500 MHz at 255 K temperature. F) 2D CC (200 ms CC transfer time) of ^13^C,^15^N‐PG in DOPC liposomes acquired at 700 MHz at 245 K temperature. G) Cross‐polarization build‐up curves measured at 1200 MHz and 17 kHz MAS using 275 K temperature show that the ^13^C‐PG headgroup is more mobile than its glycerol backbone. See Figures S6‐S8 for full assignments of labeled lipids.

While our approach is broadly applicable, in this manuscript, we show the isolation of ^13^C,^15^N‐lipids with an *anionic* cardiolipin, an *anionic* phosphatidylglycerol (PG), a *zwitterionic* phosphatidylethanolamine (PE), and a *zwitterionic* phosphatidylcholine (PC) headgroup (Figure 1A). All these lipids are involved in critical biological functions, with cardiolipin playing a central role in the respiratory chain,^[7]^ ATP synthesis,^[8]^ apoptosis,^[9]^ and mitochondrial transport,^[10]^ to name only a few examples.

## Results and Discussion

We isolated ^13^C,^15^N‐lipids from bacteria or yeast grown in isotope‐enriched tailored media (see Experimental Section and Supporting Information). Total lipids from bacteria were extracted according to the method of Bligh‐Dyer^[11]^ in the presence of lysozyme to increase cardiolipin extraction efficiency,^[12]^ while yeast lipids were extracted under mechanical disruption.^[13]^ Next, we developed a simple and fast preparative purification method, based on a single isocratic elution on silica, to purify multiple phospholipid classes from complex mixtures (see Experimental Section and Supporting Information), yielding pure fractions of different lipid classes that we analyzed by solution state NMR and gas chromatography to establish purity, lipid‐headgroup identity, and lipid‐tail composition. By comparison to synthetic standards, solution NMR data unambiguously confirmed the headgroup identity and the high purity (based on headgroup type) of our isolations (Figures S1‐4). The nature of the lipid tails varied according to the organism and is discussed further below.

Next, we performed ssNMR experiments using mixed liposomes that contained 5 mol % ^13^C,^15^N‐lipids and 95 mol% DOPC (Figure 1). Since the assignments of lipid headgroups and the glycerol backbone in membranes are heavily compromised by signal overlap, we first acquired 2D ^13^C‐^13^C PARISxy^[14]^ spectra at ultra‐high magnetic field of 1200 MHz (^1^H‐frequency) and a sample temperature of 275 K at which the investigated membranes were in the liquid‐crystalline phase with a high degree of mobility. At these experimental conditions, we could resolve all headgroup and backbone signals. This is the first time that the complete PG headgroup could be assigned in membranes. While the chemical shifts of the glycerol backbone (g1–g3) are similar for all measured lipids, we measured substantial chemical shift differences in the headgroups. Strikingly, in the liquid‐crystalline phase, most cross‐peaks in the PG and PE headgroups were not observable in dipolar spectra, which implies that the headgroups are highly flexible (Figure 1B,C,G). Indeed, by lowering the sample temperature to 255 or 245 K, at which the ^13^C,^15^N‐lipids were in the gel phase in which the mobility of individual lipids decreases sharply, we observed clear cross‐peaks for the entire PE and PG headgroups (Figure 1E,F). The unusual cardiolipin headgroup, whose mobility is severely restricted, is already fully visible in the liquid‐crystalline phase at 275 K (Figure 1D). Note that in contrast to commercially available PG, which contains a racemic glycerol headgroup,^[15]^ our purified bacterial PG‐lipids contain a glycerol headgroup in enantiomeric excess, as verified by CD spectroscopy (Figure S5).

**Figure 2:**
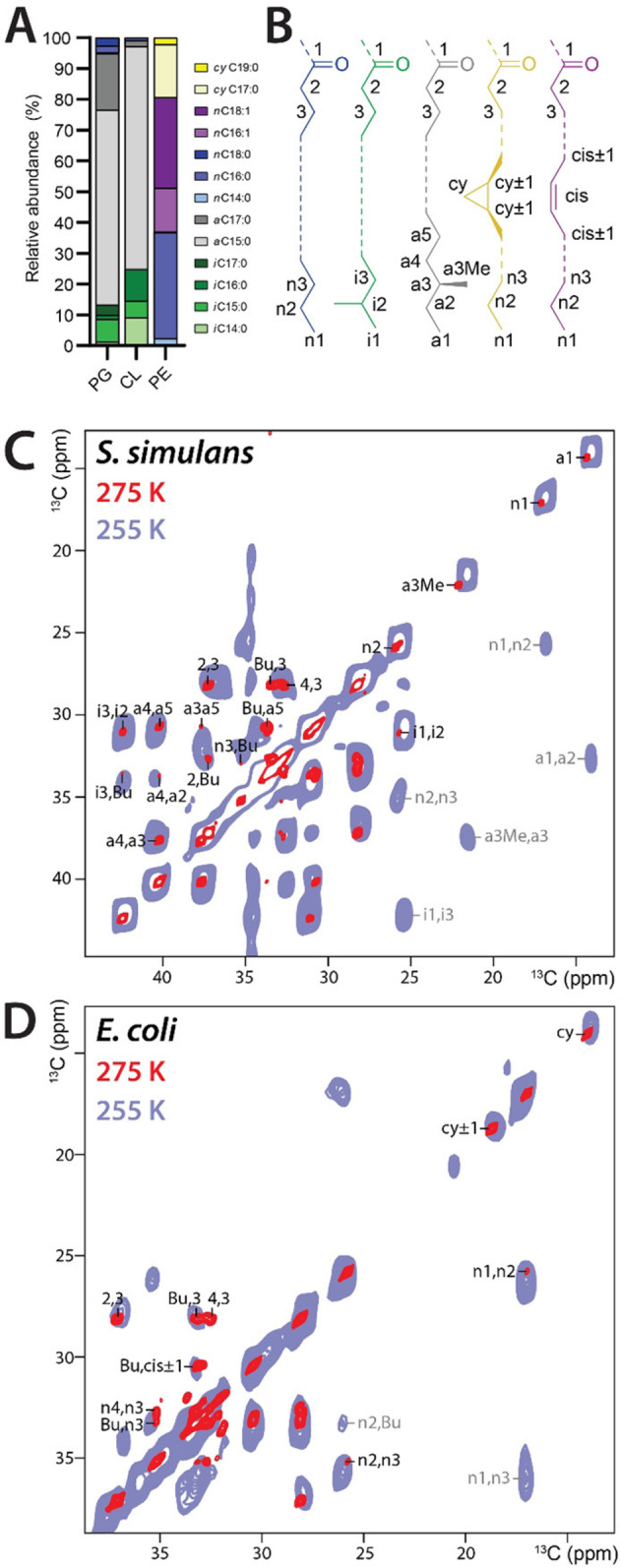
Characterization of phospholipid tails. A) Gas chromatography derived tail compositions of isolated biological ^13^C,^15^N‐lipids. B) Structures and nomenclatures of lipid tails. C) Aliphatic region of 2D CC PARISxy spectra of ^13^C‐PG‐lipids (5 mol %) from *S. simulans* in DOPC liposomes. The red spectrum was acquired at 1200 MHz and 275 K sample temperature (liquid‐crystalline phase), the blue spectrum at 500 MHz and 255 K (gel phase). D) Similar to C) but for ^13^C,^15^N‐PE‐lipids from *E. coli*. The red spectrum was acquired at 1200 MHz and 275 K, the blue spectrum at 500 MHz and 255 K.

Organisms feature a broad spectrum of lipid tails that are vital to shape membrane properties. The most well‐known example is lipid unsaturation that keeps membranes of eukaryotes fluid, but especially bacterial membranes feature a large diversity of lipid tails that include branching and cyclic moieties. This broad spectrum of lipid tails is important to fine‐tune membrane properties,^[16]^ and likely also for specific interactions with membrane proteins.^[17]^ First, we comprehensively characterized the nature of the tails using solution NMR and gas chromatography (Figure 2A,B and Figure S9). The lipid tails that were isolated from *Escherichia coli* (with a PE headgroup) were mostly straight and saturated, with 20 % of the tails containing cyclopropyl‐groups,^[5b]^ whereas lipids isolated from *Staphylococcus simulans* or *Micrococcus flavus* (with PG or cardiolipin headgroups) had tails that were fully saturated and branched (so‐called *iso* or *anteiso* methyl‐branched) at the termini.^[5a]^ In *Saccharomyces cerevisiae* (yeast), lipid tails were straight and unsaturated (Table S1 and Figure S6). We note that the composition of lipid tails can readily be altered by adjusting culture conditions such as temperature, salt concentrations, addition of detergents, or choosing a different species (Figure S10).[^5^, ^18^]

In complex liposomes, we could obtain well‐resolved 2D CC ssNMR spectra that enabled us to assign large parts of the tails with the exception of congested bulk signals (Figure 2C,D). In dipolar spectra acquired in the liquid‐crystalline phase, most correlations of the lipid‐tail termini could not be observed as the lipid mobility increases towards the membrane center. Conversely, these missing correlations involving mobile tail‐atoms could be observed in a scalar 2D CC TOBSY^[19]^ ssNMR spectrum (Figure S11). At lower temperature in the gel phase, we could observe all cross‐peaks for the tails, including for the termini, in dipolar spectra (Figures S12–13).

The available high‐resolution knowledge on the structure and the dynamics of lipid headgroups in complex liposomes is still largely derived from computational studies (see, for example, ref.^[20]^). In a similar vein, this is also the case for the molecular behavior of lipid tails in complex membranes. Thereby, ^13^C,^15^N‐lipids provide unprecedented access to molecular parameters of lipids, which we demonstrate below on the example of a lipid‐targeting drug.

An increasing number of drugs target specific phospholipids. This includes antibiotics,[^3a^, ^3b^] such as the clinically used daptomycin,^[21]^ and also antimycotics.^[3c]^ For all these drugs, due to the above‐mentioned technical challenges, the molecular details of their lipid interactions are scarce and usually elusive in membranes. As a test case, we studied cinnamycin, a 19‐residue lanthipeptide (Figure S14A) that is well‐known to target the headgroup of PE‐lipids.^[22]^ Cinnamycin is a naturally produced drug that is, in addition, used to monitor PE domains in bacterial and cancer cells,^[23]^ and has potential antiviral applications.^[24]^ A previous solution NMR structural model^[25]^ determined in DMSO using C12‐lysoPE (a truncated, soluble mimic of PE‐lipids) suggested that cinnamycin forms a binding pocket that specifically accommodates the small cationic PE headgroup, but not bulkier headgroups such as that of PC. Contacts to the phosphate group, the glycerol backbone, or even the lipid tails were not observed in DMSO. However, as outlined by a recent molecular dynamics study that observed a deep membrane penetration of cinnamycin,^[26]^ this binding model is difficult to align with experimental observations that i) cinnamycin has no affinity to PE‐sphingolipids, which have a PE headgroup but a ceramide backbone instead of a glycerol backbone,^[23]^ ii) cinnamycin is not antagonized by phosphopropanolamine^[27]^ suggesting that the distance between phosphatidylethanolamine headgroup and glycerol backbone matters, and iii) all members of the cinnamycin‐like peptide family such as duramycin have a hydrophobic stretch in common (Phe_7_‐Ala_14_), suggesting interactions with hydrophobic moieties (Figure S14B). Hence, it appears unlikely that only the PE headgroup is involved in the binding interface.

**Figure 3:**
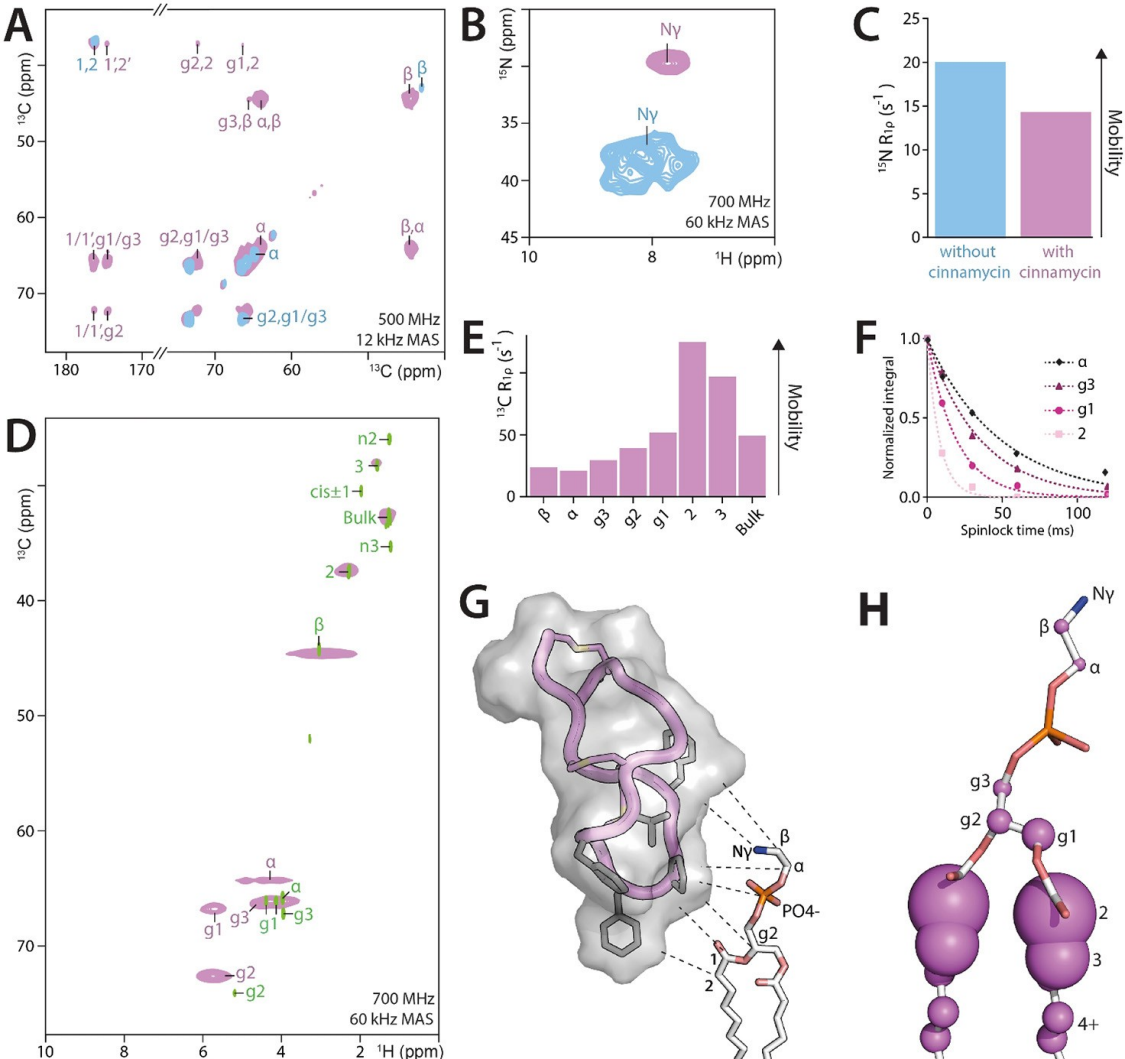
**Specific interactions between the peptide‐drug cinnamycin and PE‐lipids in mixed liposomes**. A) 2D CC PARISxy spectra of ^13^C,^15^N PE‐lipids measured in the presence (in magenta) and absence (in blue) of cinnamycin. Spectra were obtained at 500 MHz and 260 K temperature using 150 ms CC magnetization transfer. B) ^1^H‐detected 2D NH spectra acquired in the presence (in magenta) and absence (blue) of cinnamycin, obtained at 700 MHz. C) ^15^N *R*_1ρ_ relaxation rates for the ammonium‐group of the PE‐headgroup, with (magenta) and without (blue) cinnamycin. D) ^1^H‐detected 2D CH spectra of cinnamycin‐bound ^13^C,^15^N PE‐lipids (in magenta), superimposed on the solution NMR spectrum of free PE‐lipids (in green). E) ^13^C *R*_1ρ_ relaxation rates for PE‐lipids bound to cinnamycin. F) ^13^C *R*_1ρ_ signal decay curves for selected headgroup and glycerol backbone atoms. G) ssNMR studies show that not only the PE‐headgroup, but also the phosphate, the glycerol backbone, and the beginning of the lipid‐tail are part of the interface with cinnamycin. A cinnamycin orientation is shown in which conserved hydrophobic residues point towards the lipid tails. H) Illustration of the site‐resolved dynamics. The size of the spheres represents the *R*_1ρ_ relaxation rates. For all measurements, we used DOPC membranes with a small (5 mol %) fraction of ^13^C,^15^N PE‐lipids, and added an equimolar, stoichiometric amount of cinnamycin.

We prepared DOPC liposomes containing 5 mol% of ^13^C,^15^N‐PE‐lipids in the presence or the absence of 5 mol% cinnamycin and acquired 2D CC PARISxy,^[14]^ as well as ^1^H‐detected dipolar‐based 2D NH and 2D CH ssNMR spectra. Upon addition of cinnamycin, we observed large chemical shift perturbations (CSPs) for the headgroup carbons, as well as a massive (−7.4 ^15^N ppm) CSP for the NH_3_ ammonium group (Figure 3A,B,D and Figure S15). This indeed shows that the PE headgroup is tightly coordinated by cinnamycin in membranes. Furthermore, ^31^P ssNMR studies show a CSP (+1.1 ppm, Figure S16) in the presence of cinnamycin, which is in line with MD simulations that suggested hydrogen bonding with the PE‐lipid phosphate group.^[26]^

Strikingly, we also observed stark CSPs for the carbons of the glycerol backbone and the beginning of the lipid‐tail (Figure 3A). Furthermore, cross‐peaks between the beginning of the lipid tails (*sn*‐1 and *sn*‐2 carbonyl signals) and the glycerol backbone could only be observed in the presence of the drug, but not in the absence of the drug. This demonstrates that the glycerol backbone and the lipid tails stiffen in the presence of the drug. Finally, the shifts of the *sn*‐1 and *sn*‐2 carbonyl signals imply a structural change in the lipid tails. Together, these data clearly demonstrate that cinnamycin does not only bind to the PE‐headgroup, but also to the glycerol backbone and the lipid tails, i. e., an interface that strongly diverges from the structural model obtained in DMSO with truncated PE‐like lipids (Figure 3G).^[28]^ This is yet another example that a proper membrane environment is essential to obtain relevant binding modes for small molecules that target lipids in membranes.^[29]^

Finally, to quantify the observed stiffening of PE‐lipids upon target binding, we probed their dynamics by measuring atom‐resolved ^13^C and ^15^N rotating frame relaxation (R_1ρ_) data using a series of ^1^H‐detected 2D CH and 1D NH experiments (Figure 3C,E,F and Figure S17).^[30]^ ^15^N relaxation data revealed a substantial stiffening of the free ammonium‐group of the PE‐headgroup (∼20.1 ms ^15^N R_1ρ_ in the absence of cinnamycin; ∼14.4 ms ^15^N R_1ρ_ in the presence of cinnamycin). Strikingly, in the presence of cinnamycin, ^13^C relaxation data show a marked stiffening of the entire PE‐headgroup (∼20 ms ^13^C R_1ρ_) that is substantially more rigid than the glycerol backbone (∼40 ms ^13^C R_1ρ_) (Figure 3H). This is in stark contrast to the dynamics of free PE‐lipids, where the headgroup is highly mobile (Figure 1B). Note that the headgroup of free PE‐lipids gave only weak and broad dipolar signals in 2D CH spectra at 60 kHz MAS and a sample temperature of approximately 310 K, presumably because of strong dynamics that interfered with magnetization transfer (data not shown).

## Conclusions

Here, we have introduced an ssNMR approach that enables detailed high‐resolution insights into phospholipids in complex fluid membranes. As shown on the example of the peptide drug cinnamycin, this broadly applicable approach provides new vistas for the characterization of small molecules, drugs, or proteins that specifically interact with phospholipids. In particular, the combined sensitivity gains of ^13^C,^15^N‐lipids and modern ^1^H‐detected methods at fast sample spinning enable detailed multi‐dimensional relaxation studies in mixed liposomes with traces (∼5 mol%) of the lipid of interest. Thereby, our approach opens new opportunities to characterize the molecular properties of phospholipids and their interactions in biologically relevant membranes. Previously, ssNMR studies of specific lipids in complex membranes were usually limited to either the phosphorus as the sole reporter^[31]^ or to rare, serendipitous events of co‐purified endogenous ^13^C,^15^N‐lipids.[^4g^, ^4h^, ^32^] The here introduced approach is also complementary to the use of deuterated phospholipids, which are commercially available and are widely used to study microsecond motion of lipids using static ssNMR spectroscopy.^[33]^ We note that our labelling strategy can be further finetuned by using specifically labeled precursors to reduce spectral crowding, which seems especially useful to resolve spectral congestion of the lipid headgroup/glycerol backbone signals. As proof‐of‐principle we grew bacteria on 1‐^13^C_1_‐D‐Glc or 2‐^13^C_1_‐D‐Glc,^[34]^ yielding a specifically labeled PE‐lipids (Figure S18). Such a sparse labelling strategy could be valuable to probe specific lipid binding to membrane proteins, as recently shown on the example of sterol‐lipids.^[35]^

## Experimental Section

### Strains

Staphylococcus simulans 22, Micrococcus flavus DSM 1790, Escherichia coli BL21(DE3), Bacillus cereus ATCC 10987, and Saccharomyces cerevisiae BY4742 were used in this work.

**Cell growth**: Bacteria were revived from glycerol stocks on fresh plates of LB 1.8 w/v% agar (*S. simulans*, *E. coli*, *B. cereus*) or TSB 1.8 w/v% agar (*M. flavus*) for 24 h at either 30 °C (*M. flavus*, *B. cereus*) or 37 °C (*S. simulans*, *E. coli*). From this fresh plate, a single colony was inoculated in an overnight culture (50 mL in a 250 mL unbaffled flask) in the same medium without agar. The medium was removed by centrifugation (4,000×*g*, 10 min, RT), and the bacterial pellets resuspended to OD_600_=0.05 in 1 L of medium A (Table S2) in a 2 L baffled flask. Bacteria were grown at either 30 °C or 37 °C as before, with 200 or 220 RPM shaking respectively and harvested by centrifugation (4,000×*g*, 4 °C, 15–30 min) at late‐log phase.

*Sa. cerevisiae* was grown in Standard Defined (SD) medium (Table S3). Glycerol stocks were revived on a SD 1.8 w/v% agar plate for 48 h at 30 °C. Single colonies were inoculated into liquid SD medium (50 mL in a 250 mL unbaffled flask) and grown overnight. Finally, precultures were diluted in 1 L medium B (Table S4) in a 2 L baffled flask to OD_600_=0.05 and grown for 40 h at 30 °C before harvesting by centrifugation.

**Lipid isolation**: Bacteria were resuspended in 250 mM phosphate buffer (pH=7.4) to an OD_600_ of ∼50, after which they were treated with lysozyme and incubated at 37 °C for 2 h with shaking. Subsequently, two volumes of MeOH and one volume of CHCl_3_ were added and the resulting suspension was stirred vigorously for 30 minutes at RT. Then, one volume of CHCl_3_ and 0.8 volumes of H_2_O were added, and the organic phase was collected and evaporated to dryness. Dried lipid films were resuspended in CHCl_3_ assisted by sonication and loaded on a silica column equilibrated in CHCl_3_ (silica 60, 0.040–0.063 mm). The column was washed with CHCl_3_ and phospholipids were eluted with CHCl_3_/EtOH/25 %NH_3_/H_2_O 48 : 48 : 1 : 3 (v/v/v/v)+0.2 g/L NH_4_Ac. Fractions were analyzed on TLC using the same eluent (HPTLC Nano‐ADAMANT® 0.20 mm silica 60, stained by iodine vapor and/or molybdenum blue^[36]^). Pure fractions were combined, evaporated and redissolved in 2 : 1 CHCl_3_/MeOH (v/v). Small quantities of phospholipids were isolated using preparative TLC. 5 mm horizontal lanes of lipid extracts in 2 : 1 CHCl_3_/MeOH (v/v) were spotted using a CAMAG® Linomat 5 semi‐automatic sample application system and developed in CHCl_3_/EtOH/25 %NH_3_/H_2_O 48 : 48 : 1 : 3 (v/v/v/v)+0.2 g/L NH_4_Ac. TLC plates were dried under a flow of N_2_, after which a small vertical lane was cut and stained with iodine vapor to identify lipid spots. Horizontal lanes of interest were scratched off and lipids were extracted from the silica by extensive vortexing in 2 : 1 CHCl_3_/MeOH (v/v) followed by centrifugation (4,700×*g*). Silica was extracted thrice to ensure quantitative transfer.

For *Saccharomyces cerevisiae* lipids a different extraction procedure was used. Lyophilized cells were resuspended in 2 : 1 CHCl_3_/50 mM HCl (v/v) and subjected to multiple sonication (Branson 3800) and stirring cycles, followed by filtration of the suspension over a glass filter (porosity index 0.3). The organic phase was then collected, evaporated to dryness, and the residue purified as described above for bacteria.

We obtained typical yields of >10 mg of the highest abundant lipid class of the microorganism of choice at >99 % purity based on solution NMR analysis.

**Solid state NMR sample preparation**: Appropriate volumes of lipid stocks in CHCl_3_ or CHCl_3_/MeOH were mixed, evaporated under a stream of N_2_ gas and exposed to high vacuum for 20 minutes. The resulting lipid films were resuspended in buffer (50 mM HEPES, 150 mM NaCl, pH=7.2) by extensive vortexing. The formed lipid vesicles were then collected by ultra‐centrifugation (100k×g, 4 °C, 30 min) and spun down into 3.2 or 1.3 mm solid state NMR rotors. Samples containing cinnamycin were made by including cinnamycin (at 1 : 1 U‐[^13^C,^15^N]‐PE to cinnamycin stoichiometry) in the resuspension buffer.

**NMR spectroscopy**: Solution state NMR spectra of isolated phospholipid and appropriate commercial standards were recorded in 2 : 1 CD_3_OD/CDCl_3_ (v/v) at 298 K and at 14.1 T (600 MHz ^1^H frequency). Chemical shifts were referenced to DSS using internal TMS. Assignments were done using standard experiments such as HSQC, COSY, TOCSY, HMBC on the lipid standards and compared to the literature (e. g. BMRB^[37]^ entry bmse001105). For purified U‐[^13^C,^15^N] enriched lipids assignments were copied from the standards or literature^[38]^ and confirmed with 2D ^1^H‐^13^C‐CT‐HSQC‐TOCSY experiments.

Solid state NMR spectra were recorded at 11.7, 16.4, or 28.2 T (500, 700, and 1200 MHz ^1^H frequency, respectively). Dipolar based 2D ^13^C‐^13^C spin diffusion experiments were acquired with PARISxy^[14]^ recoupling (m=1) and SPINAL64^[39]^ decoupling. At 1200 MHz we used 17 kHz MAS and a mixing time of 200 ms, at 700 MHz we used 10 kHz MAS and a mixing time of 200 ms, and at 500 MHz we used 12 kHz MAS and a mixing time of 150 ms. TOBSY^[19]^ scalar coupling‐based 2D ^13^C^13^C experiments were conducted at 305 K and 8 kHz MAS with a mixing time of 6 ms. An INEPT block was used for the initial ^1^H‐^13^C magnetization transfer and WALTZ16 decoupling was applied in both dimensions. ^31^P spectra were recorded at 11.7 T (500 MHz ^1^H frequency) and 10 kHz MAS and a contact time of 1.5 ms using SPINAL64^[39]^ decoupling. ^1^H‐detected experiments were performed at 60 kHz MAS and 16.4 T (700 MHz ^1^H frequency) using low‐power PISSARRO^[40]^ decoupling in all dimensions and a sample temperature of 305 K. ^1^H‐detected R_1rho_ relaxation experiments were acquired with a ^15^N/^13^C spin‐lock field of 18 kHz and durations of 0, 10, 25, 50 and 75 ms for ^15^N and 0, 10, 30, 60 and 120 ms for ^13^C. Chemical shifts were referenced externally using adamantane (CH at 31.48 ppm). Effective sample temperatures were calibrated using the ^79^Br resonance of KBr as previously described.^[41]^

**Gas chromatography (GC)**: Dried phospholipids (0.2‐0.5 mg) were redissolved in 1 mL *n*‐hexane by extensive shaking, followed by the addition of 200 μL 100 g/L KOH in MeOH. Samples were vigorously shaken for at least 1 min to mix both layers well. The *n*‐hexane layer was then taken and dried under a flow of N_2_ at 40 °C. The obtained fatty acid methyl esters (FAMEs) were redissolved in 50 μL *n*‐hexane and analyzed using gas chromatography with flame‐ionization detection on a Trace GC Ultra (Thermo Fisher Scientific) equipped with a 30 m long biscyanopropyl polysiloxane column (internal diameter 0.25 mm; Restek) and N_2_ as the carrier gas. A temperature gradient was applied, starting at 40 °C, held for one minute, then increasing linearly to 160 °C in 4 min and finally to 220 °C in 15 min. Peaks were identified using FAME standards Mixture BR2 (Larodan; 90‐1052) for branched species and certain straight chain fatty acids or 63‐B (Nu‐Chek‐Prep) for various unsaturated and straight chain fatty acids. Assignments not included in these mixtures were made based on expected elution patterns that could be deduced from the standards. FAME‐species that are noted as “unidentified” in this work elute after *n*C18:0.

**Circular dichroism (CD) spectroscopy**: CD spectroscopy was performed on a Jasco J‐810‐150S spectropolarimeter, using a 1 mm quartz cuvette at 20 °C. Lipids were dissolved to 1 g/L in MeOH. Obtained spectra were corrected for a MeOH blank. Spectra were averaged over five scans and represent data for which the absorbance was ≤2.0.

## Conflict of interest

The authors declare no conflict of interest.

1

## Supporting information

As a service to our authors and readers, this journal provides supporting information supplied by the authors. Such materials are peer reviewed and may be re‐organized for online delivery, but are not copy‐edited or typeset. Technical support issues arising from supporting information (other than missing files) should be addressed to the authors.

Supporting Information

## Data Availability

The data that support the findings of this study are available from the corresponding author upon reasonable request. The solution and solid‐state NMR assignments of lipids have been deposited in the BMRbig (https://bmrbig.bmrb.io/) Data Bank (accession codes BMRbig75, BMRbig76, BMRbig77, BMRbig78,  BMRbig79, BMRbig80, BMRbig81).

## References

[1a] A. Laganowsky , E. Reading , T. M. Allison , M. B. Ulmschneider , M. T. Degiacomi , A. J. Baldwin , C. V. Robinson , Nature 2014, 510, 172–175; 24899312 10.1038/nature13419 PMC4087533

[1b] R. Amani , C. G. Borcik , N. H. Khan , D. B. Versteeg , M. Yekefallah , H. Q. Do , H. R. Coats , B. J. Wylie , Proc. Natl. Acad. Sci. USA 2020, 117, 2938–2947; 31980523 10.1073/pnas.1915010117 PMC7022178

[1c] S. B. Hansen , X. Tao , R. MacKinnon , Nature 2011, 477, 495–498; 21874019 10.1038/nature10370 PMC3324908

[1d] J. E. Harlan , P. J. Hajduk , H. S. Yoon , S. W. Fesik , Nature 1994, 371, 168–170. 8072546 10.1038/371168a0

[2a] A. G. Lee , Biochim. Biophys. Acta Biomembr. 2003, 1612, 1–40; 10.1016/s0005-2736(03)00056-7 12729927

[2b] H. I. Ingólfsson , M. N. Melo , F. J. van Eerden , C. Arnarez , C. A. Lopez , T. A. Wassenaar , X. Periole , A. H. de Vries , D. P. Tieleman , S. J. Marrink , J. Am. Chem. Soc. 2014, 136, 14554–14559; 25229711 10.1021/ja507832e

[2c] E. Mileykovskaya , W. Dowhan , Biochim. Biophys. Acta Biomembr. 2009, 1788, 2084–2091; 10.1016/j.bbamem.2009.04.003 PMC2757463 19371718

[2d] M. P. Czech , Cell 2000, 100, 603–606. 10761925 10.1016/s0092-8674(00)80696-0

[3a] C. W. Johnston , M. A. Skinnider , C. A. Dejong , P. N. Rees , G. M. Chen , C. G. Walker , S. French , E. D. Brown , J. Berdy , D. Y. Liu , N. A. Magarvey , Nat. Chem. Biol. 2016, 12, 233–239; 26829473 10.1038/nchembio.2018

[3b] M. Song , Y. Liu , X. Huang , S. Ding , Y. Wang , J. Shen , K. Zhu , Nat. Microbiol. 2020, 5, 1040–1050; 32424338 10.1038/s41564-020-0723-z

[3c] M. Järvå , F. T. Lay , T. K. Phan , C. Humble , I. K. H. Poon , M. R. Bleackley , M. A. Anderson , M. D. Hulett , M. Kvansakul , Nat. Commun. 2018, 9. 10.1038/s41467-018-04434-y PMC5958116 29773800

[4a] J. M. Boettcher , R. L. Davis-Harrison , M. C. Clay , A. J. Nieuwkoop , Y. Z. Ohkubo , E. Tajkhorshid , J. H. Morrissey , C. M. Rienstra , Biochemistry 2011, 50, 2264–2273; 21294564 10.1021/bi1013694 PMC3069658

[4b] A. A. Smith , A. Vogel , O. Engberg , P. W. Hildebrand , D. Huster , Nat. Commun. 2022, 13; 10.1038/s41467-021-27417-y PMC8748619 35013165

[4c] M. Li , A. Mandal , V. A. Tyurin , M. DeLucia , J. Ahn , V. E. Kagan , P. C. A. van der Wel , Structure 2019, 27, 806–815.e804; 30879887 10.1016/j.str.2019.02.007 PMC6615723

[4d] V. S. Mandala , J. K. Williams , M. Hong , Annu. Rev. Biophys. 2018, 47, 201–222; 29498890 10.1146/annurev-biophys-070816-033712 PMC6312106

[4e] E. A. W. van der Cruijsen , D. Nand , M. Weingarth , A. Prokofyev , S. Hornig , A. A. Cukkemane , A. M. J. J. Bonvin , S. Becker , R. E. Hulse , E. Perozo , O. Pongs , M. Baldus , Proc. Natl. Acad. Sci. USA 2013, 110, 13008–13013; 23882077 10.1073/pnas.1305563110 PMC3740848

[4f] M. T. Eddy , T.-C. Ong , L. Clark , O. Teijido , P. C. A. van der Wel , R. Garces , G. Wagner , T. K. Rostovtseva , R. G. Griffin , J. Am. Chem. Soc. 2012, 134, 6375–6387; 22435461 10.1021/ja300347v PMC3333839

[4g] J. E. de Vlugt , P. Xiao , R. Munro , A. Charchoglyan , D. Brewer , M. S. Al-Abdul-Wahid , L. S. Brown , V. Ladizhansky , Biochim. Biophys. Acta Biomembr. 2020, 1862; 10.1016/j.bbamem.2020.183345 32407777

[4h] P. Hariharan , E. Tikhonova , J. Medeiros-Silva , A. Jeucken , M. V. Bogdanov , W. Dowhan , J. F. Brouwers , M. Weingarth , L. Guan , BMC Biol. 2018, 16. 10.1186/s12915-018-0553-0 PMC6091025 30075778

[5a] T. Kaneda , Microbiol. Rev. 1991, 55, 288–302; 1886522 10.1128/mr.55.2.288-302.1991 PMC372815

[5b] V. A. Knivett , J. Cullen , Biochem. J. 1965, 96, 771–776. 5324304 10.1042/bj0960771 PMC1207215

[6a] V. Chevelkov , K. Rehbein , A. Diehl , B. Reif , Angew. Chem. Int. Ed. 2006, 45, 3878–3881; 10.1002/anie.200600328 16646097

[6b] D. H. Zhou , G. Shah , M. Cormos , C. Mullen , D. Sandoz , C. M. Rienstra , J. Am. Chem. Soc. 2007, 129, 11791–11801; 17725352 10.1021/ja073462m

[6c] V. Agarwal , S. Penzel , K. Szekely , R. Cadalbert , E. Testori , A. Oss , J. Past , A. Samoson , M. Ernst , A. Böckmann , B. H. Meier , Angew. Chem. Int. Ed. 2014, 53, 12253–12256; 10.1002/anie.201405730 25225004

[6d] L. B. Andreas , K. Jaudzems , J. Stanek , D. Lalli , A. Bertarello , T. Le Marchand , D. Cala-De Paepe , S. Kotelovica , I. Akopjana , B. Knott , S. Wegner , F. Engelke , A. Lesage , L. Emsley , K. Tars , T. Herrmann , G. Pintacuda , Proc. Natl. Acad. Sci. USA 2016, 113, 9187–9192; 27489348 10.1073/pnas.1602248113 PMC4995937

[6e] J. Medeiros-Silva , D. Mance , M. Daniëls , S. Jekhmane , K. Houben , M. Baldus , M. Weingarth , Angew. Chem. Int. Ed. 2016, 55, 13606–13610; 10.1002/anie.201606594 PMC5113794 27671832

[6f] E. E. Najbauer , K. Tekwani Movellan , K. Giller , R. Benz , S. Becker , C. Griesinger , L. B. Andreas , J. Am. Chem. Soc. 2022, 144, 2953–2967. 35164499 10.1021/jacs.1c09848 PMC8874904

[7] K. Pfeiffer , V. Gohil , R. A. Stuart , C. Hunte , U. Brandt , M. L. Greenberg , H. Schägger , J. Biol. Chem. 2003, 278, 52873–52880. 14561769 10.1074/jbc.M308366200

[8] A. Mühleip , S. E. McComas , A. Amunts , eLife 2019, 8. 10.7554/eLife.51179 PMC6930080 31738165

[9] J. B. McMillin , W. Dowhan , Biochim. Biophys. Acta - Mol. Cell Biol. Lipids 2002, 1585, 97–107. 10.1016/s1388-1981(02)00329-3 12531542

[10a] S. Ghosh , W. Basu Ball , T. R. Madaris , S. Srikantan , M. Madesh , V. K. Mootha , V. M. Gohil , Proc. Natl. Acad. Sci. USA 2020, 117, 16383–16390; 32601238 10.1073/pnas.2000640117 PMC7368250

[10b] K. Malhotra , A. Modak , S. Nangia , T. H. Daman , U. Gunsel , V. L. Robinson , D. Mokranjac , E. R. May , N. N. Alder , Sci. Adv. 2017, 3. 10.1126/sciadv.1700532 PMC5580885 28879236

[11] E. G. Bligh , W. J. Dyer , Can. J. Biochem. Physiol. 1959, 37, 911–917. 13671378 10.1139/o59-099

[12] M. H. Filgueiras , J. A. F. O. den Kamp , Biochim. Biophys. Acta, Lipids Lipid Metab. 1980, 620, 332–337. 10.1016/0005-2760(80)90215-5 6776994

[13] R. Letters , Biochim. Biophys. Acta, Lipids Lipid Metab. 1966, 116, 489–499. 5963012

[14] M. Weingarth , G. Bodenhausen , P. Tekely , Chem. Phys. Lett. 2010, 488, 10–16.

[15] P. D′Arrigo , L. de Ferra , G. Pedrocchi-Fantoni , D. Scarcelli , S. Servi , A. Strini , J. Chem. Soc. Perkin Trans. 1 1996, 2657–2660.

[16] K. Pluhackova , A. Horner , BMC Biol. 2021, 19. 10.1186/s12915-020-00936-8 PMC7807449 33441107

[17a] C. Pliotas , A. C. E. Dahl , T. Rasmussen , K. R. Mahendran , T. K. Smith , P. Marius , J. Gault , T. Banda , A. Rasmussen , S. Miller , C. V. Robinson , H. Bayley , M. S. P. Sansom , I. R. Booth , J. H. Naismith , Nat. Struct. Mol. Biol. 2015, 22, 991–998; 26551077 10.1038/nsmb.3120 PMC4675090

[17b] K. Gupta , J. A. C. Donlan , J. T. S. Hopper , P. Uzdavinys , M. Landreh , W. B. Struwe , D. Drew , A. J. Baldwin , P. J. Stansfeld , C. V. Robinson , Nature 2017, 541, 421–424. 28077870 10.1038/nature20820 PMC5501331

[18] W. M. O′Leary , S. G. Wilkinson , in Microbial Lipids, Vol. 2 (Eds.: C. Ratledge , S. G. Wilkinson ), Academic Press, 1988, pp. 117–201.

[19] M. Baldus , B. H. Meier , J. Magn. Reson. Ser. A 1996, 121, 65–69.

[20] G. Hedger , M. S. P. Sansom , Biochim. Biophys. Acta Biomembr. 2016, 1858, 2390–2400. 10.1016/j.bbamem.2016.02.037 PMC5589069 26946244

[21] I. Kotsogianni , T. M. Wood , F. M. Alexander , S. A. Cochrane , N. I. Martin , ACS Infect. Dis. 2021, 7, 2612–2619. 34406007 10.1021/acsinfecdis.1c00316 PMC8438661

[22] G. Machaidze , J. Seelig , Biochemistry 2003, 42, 12570–12576. 14580203 10.1021/bi035225b

[23] F. Hullin-Matsuda , A. Makino , M. Murate , T. Kobayashi , Biochimie 2016, 130, 81–90. 27693589 10.1016/j.biochi.2016.09.020

[24] N. Naruse , O. Tenmyo , K. Tomita , M. Konishi , T. Miyaki , H. Kawaguchi , K. Fukase , T. Wakamiya , T. Shiba , J. Antibiot. 1989, 42, 837–845. 10.7164/antibiotics.42.837 2544544

[25] K. Hosoda , M. Ohya , T. Kohno , T. Maeda , S. Endo , K. Wakamatsu , Journal of Biochemistry 1996, 119, 226–230. 8882709 10.1093/oxfordjournals.jbchem.a021226

[26] M. Vestergaard , N. A. Berglund , P.-C. Hsu , C. Song , H. Koldsø , B. Schiøtt , M. S. P. Sansom , ACS Omega 2019, 4, 18889–18899. 31737850 10.1021/acsomega.9b02949 PMC6854821

[27] S.-Y. Choung , T. Kobayashi , J.-i. Inoue , K. Takemoto , H. Ishitsuka , K. Inoue , Biochim. Biophys. Acta Biomembr. 1988, 940, 171–179. 10.1016/0005-2736(88)90192-7 3370206

[28] K. Wakamatsu , S. Y. Choung , T. Kobayashi , K. Inoue , T. Higashijima , T. Miyazawa , Biochemistry 1990, 29, 113–118. 2157477 10.1021/bi00453a013

[29a] J. Medeiros-Silva , S. Jekhmane , A. L. Paioni , K. Gawarecka , M. Baldus , E. Swiezewska , E. Breukink , M. Weingarth , Nat. Commun. 2018, 9; 10.1038/s41467-018-06314-x PMC6160437 30262913

[29b] R. Shukla , J. Medeiros-Silva , A. Parmar , B. J. A. Vermeulen , S. Das , A. L. Paioni , S. Jekhmane , J. Lorent , A. M. J. J. Bonvin , M. Baldus , M. Lelli , E. J. A. Veldhuizen , E. Breukink , I. Singh , M. Weingarth , Nat. Commun. 2020, 11; 10.1038/s41467-020-16600-2 PMC7275090 32503964

[29c] R. Shukla , F. Lavore , S. Maity , M. G. N. Derks , C. R. Jones , B. J. A. Vermeulen , A. Melcrová , M. A. Morris , L. M. Becker , X. Wang , R. Kumar , J. Medeiros-Silva , R. A. M. van Beekveld , A. M. J. J. Bonvin , J. H. Lorent , M. Lelli , J. S. Nowick , H. D. MacGillavry , A. J. Peoples , A. L. Spoering , L. L. Ling , D. E. Hughes , W. H. Roos , E. Breukink , K. Lewis , M. Weingarth , Nature 2022, 608, 390–396. 35922513 10.1038/s41586-022-05019-y PMC9365693

[30a] J. R. Lewandowski , H. J. Sass , S. Grzesiek , M. Blackledge , L. Emsley , J. Am. Chem. Soc. 2011, 133, 16762–16765; 21923156 10.1021/ja206815h

[30b] S. Jekhmane , J. Medeiros-Silva , J. Li , F. Kümmerer , C. Müller-Hermes , M. Baldus , B. Roux , M. Weingarth , Nat. Commun. 2019, 10. 10.1038/s41467-018-07973-6 PMC6328603 30631074

[31] D. E. Warschawski , A. A. Arnold , I. Marcotte , Biophys. J. 2018, 114, 1368–1376. 29590594 10.1016/j.bpj.2018.01.025 PMC5883613

[32a] S. Laage , Y. Tao , A. E. McDermott , Biochim. Biophys. Acta Biomembr. 2015, 1848, 260–265; 10.1016/j.bbamem.2014.08.021 PMC5526087 25168468

[32b] M. Weingarth , A. Prokofyev , E. A. W. van der Cruijsen , D. Nand , A. M. J. J. Bonvin , O. Pongs , M. Baldus , J. Am. Chem. Soc. 2013, 135, 3983–3988; 23425320 10.1021/ja3119114

[32c] M. E. Ward , E. Ritz , M. A. M. Ahmed , V. V. Bamm , G. Harauz , L. S. Brown , V. Ladizhansky , J. Biomol. NMR 2015, 63, 375–388. 26494649 10.1007/s10858-015-9997-5

[33a] A. Legrand , D. Martinez , A. Grélard , M. Berbon , E. Morvan , A. Tawani , A. Loquet , S. Mongrand , B. Habenstein , Front. Mol. Biosci. 2019, 6; 10.3389/fmolb.2019.00107 PMC6803476 31681795

[33b] J. J. Kinnun , K. J. Mallikarjunaiah , H. I. Petrache , M. F. Brown , Biochim. Biophys. Acta - Biomembranes 2015, 1848, 246–259; 10.1016/j.bbamem.2014.06.004 PMC5233721 24946141

[33c] R. Dazzoni , A. Grélard , E. Morvan , A. Bouter , C. J. Applebee , A. Loquet , B. Larijani , E. J. Dufourc , Sci. Rep. 2020, 10. 10.1038/s41598-020-61746-0 PMC7083927 32198481

[34] A. Loquet , K. Giller , S. Becker , A. Lange , J. Am. Chem. Soc. 2010, 132, 15164–15166. 20932028 10.1021/ja107460j

[35a] C. G. Borcik , I. R. Eason , M. Yekefallah , R. Amani , R. Han , B. H. Vanderloop , B. J. Wylie , Angew. Chem. Int. Ed. 2022, 61; 10.1002/anie.202112232 PMC8957755 34985791

[35b] M. R. Elkins , I. V. Sergeyev , M. Hong , J. Am. Chem. Soc. 2018, 140, 15437–15449; 30338997 10.1021/jacs.8b09658 PMC6361393

[35c] M. R. Elkins , J. K. Williams , M. D. Gelenter , P. Dai , B. Kwon , I. V. Sergeyev , B. L. Pentelute , M. Hong , Proc. Natl. Acad. Sci. USA 2017, 114, 12946–12951; 29158386 10.1073/pnas.1715127114 PMC5724280

[35d] T. M. Anderson , M. C. Clay , A. G. Cioffi , K. A. Diaz , G. S. Hisao , M. D. Tuttle , A. J. Nieuwkoop , G. Comellas , N. Maryum , S. Wang , B. E. Uno , E. L. Wildeman , T. Gonen , C. M. Rienstra , M. D. Burke , Nat. Chem. Biol. 2014, 10, 400–406. 24681535 10.1038/nchembio.1496 PMC3992202

[36] J. C. Dittmer , R. L. Lester , J. Lipid Res. 1964, 5, 126–127. 14173318

[37] E. L. Ulrich , H. Akutsu , J. F. Doreleijers , Y. Harano , Y. E. Ioannidis , J. Lin , M. Livny , S. Mading , D. Maziuk , Z. Miller , E. Nakatani , C. F. Schulte , D. E. Tolmie , R. Kent Wenger , H. Yao , J. L. Markley , Nucleic Acids Res. 2008, 36, D402–408. 17984079 10.1093/nar/gkm957 PMC2238925

[38a] G. Knothe , Lipids 2006, 41, 393–396; 16808153 10.1007/s11745-006-5110-x

[38b] F. D. Gunstone , Chem. Phys. Lipids 1993, 65, 155–163.

[39] B. M. Fung , A. K. Khitrin , K. Ermolaev , J. Magn. Reson. 2000, 142, 97–101. 10617439 10.1006/jmre.1999.1896

[40] M. Weingarth , G. Bodenhausen , P. Tekely , J. Magn. Reson. 2009, 199, 238–241. 19467891 10.1016/j.jmr.2009.04.015

[41] K. R. Thurber , R. Tycko , J. Magn. Reson. 2009, 196, 84–87. 18930418 10.1016/j.jmr.2008.09.019 PMC2632797

