# Supplementary material for: Specific Lipid Studies in Complex Membranes by Solid‐State NMR Spectroscopy
Source: Chemistry. 2022 Oct 25;28(70):e202202472. doi: 10.1002/chem.202202472 (PMC10092488; doi:10.1002/chem.202202472)
Supplement: Supplementary file 1 — Supporting Information [file CHEM-28-0-s001.pdf]

Chemistry–A European Journal

Supporting Information

Specific Lipid Studies in Complex Membranes by Solid-State NMR Spectroscopy

Roy A. M. van Beekveld, Maik G. N. Derks, Raj Kumar, Leanna Smid, Thorben Maass, João Medeiros-Silva, Eefjan Breukink, and Markus Weingarth*

Supporting Information

Table S1: List of isolated phospholipids

Lipid class	Source(s)	Lipid tails
Phosphatidyl glycerol (**PG**)	*M. flavus*	Saturated; straight, iso and anteiso
	S. simulans	Saturated; straight, iso and anteiso
Cardiolipin (**CL**)	*M. flavus*	Saturated; straight, iso and anteiso
	S. simulans	Saturated; straight, iso and anteiso
Phosphatidyl ethanolamine (**PE**)	*E. coli*	Saturated, unsaturated, cyclo-propyl
	B. cereus	Saturated; straight iso and anteiso
Phosphatidyl choline (**PC**)	*Sa. cerevisiae*	Saturated, unsaturated

Table S2: Composition of Medium A

Component	Concentration
$^{13}C^{15}N$ Silantes® OD2 rich medium	60 v%
$^{13}C^{15}N$ Bioexpress® (10x)	0.5 v%
^{13}C-Glucose	2 g/L
$^{15}NH_4Cl$	1 g/L
M9 minimal medium	39.5 v%

Table S3: Composition of Standard Defined (SD) medium

Component	Concentration
D-Glucose	20 g/L
Yeast Nitrogen Base w/o Amino Acids	6.7 g/L
$(NH_4)_2SO_4$	5 g/L
L-Leu	60 mg/L
Uracil	40 mg/L
L-Lys	30 mg/L
L-His	20 mg/L

Table S4: Composition of medium B

Component	Concentration
^{13}C-D-Glucose	2 g/L
Yeast Nitrogen Base w/o Amino Acids and Ammonium Sulfate	1.7 g/L
$(^{15}NH_4)_2SO_4$	5 g/L
$^{13}C^{15}N$ Silantes® OD2 rich medium	10 v%
$^{13}C^{15}N$ Bioexpress® (10x)	0.5 v%
L-Lys	60 mg/L

L-Leu	120 mg/L
L-His	40 mg/L
Uracil	80 mg/L

Table S5: Solution state NMR chemical shift assignments of representative isolated $^{13}C^{15}N$ enriched phospholipids.

	**$^{13}C^{15}N$ PE**		**$^{13}C^{15}N$ PG**		**$^{13}C^{15}N$ CL**		**$^{13}C^{15}N$ PC**	
	E. coli		***S. simulans***		***S. simulans***		***Sa. cerevisiae***	
	**^{13}C**	**^{1}H**	**^{13}C**	**^{1}H**	**^{13}C**	**^{1}H**	**^{13}C**	**^{1}H**
**g1**	66.01	4.19<br>4.45	65.60	4.19<br>4.41	65.81	4.20<br>4.43	66.07	4.17<br>4.44
**g2**	74.02	5.24	73.50	5.26	73.72	5.25	74.01	5.24
**g3**	67.12	4.01	68.51	4.17	67.31	4.05	67.13	4.00
**α**	65.79	4.02	71.63	4.000<br>4.06	69.86	4.02	62.64	4.26
**β**	44.23	3.10	74.22	3.83	72.97	3.96	69.83	3.62
**γ**			65.98	3.60			57.21	3.23
**2**	37.51	2.34	37.56	2.35	37.53	2.34	37.44	2.33
**3**	28.33	1.62	28.41	1.62	28.36	1.61	28.32	1.62
**Bulk**	32.65	1.33	32.86	1.31	33.00	1.33	33.00	1.29
cis	133.3	5.34					133.3	5.34
***cis*±1**	30.55	2.03					30.53	2.03
**cy**	14.09	0.59[a]<br>-0.32[b]						
**cy±1**	19.16	0.67						
**cy±2**	32.24	1.17						
**n1**	17.07	0.90	17.16	0.89	16.07	0.89	17.06	0.90
**n2**	26.06	1.32	26.17	1.31	26.07	1.31	26.06	1.31
**n3**	35.37	1.28	35.49	1.27	35.43	1.28	35.31	1.28
**i1**			25.75	0.87	25.69	0.87		
**i2**			31.52	1.52	31.42	1.52		
**i3**			42.65	1.16	42.56	1.17		
**a1**			14.47	0.87	14.39	0.87		
**a2**			33.02	1.14	32.94	1.14		
**a3**			38.04	1.30	37.92	1.30		
**a4**			40.23	1.09<br>1.29	40.13	1.09<br>1.30		
**a5**			30.96	1.28	30.78	1.28		

[a] H-*trans* [b] H-*cis*

Table S6: Solid state NMR chemical shift assignments. 5 mol% [^{13}C,^{15}N]-lipids in DOPC at 275 K.

	**$^{13}C^{15}N$ PE**	**$^{13}C^{15}N$ PG**	**$^{13}C^{15}N$ CL**
	E. coli	***S. simulans***	***M. flavus***
	^{13}C	***^{13}C***	***^{13}C***
**g1**	66.13	66.22	66.14
**g2**	73.66	73.73	73.77
**g3**	66.75	66.73	66.73
**α**	65.07	69.72	69.52

**β**	43.4	74.15	72.81
**γ**		66.22	
**1**	176.46	176.61	176.51
**2**	37.11	37.23	37.38
**3**	28.13	28.27	28.30
**Bulk**	~33.2	~33.2	~33.2
cis	132.66		
***cis*±1**	30.47		
**cy**	14.06		
**cy±1**	18.75		
**n1**	17.02	17.18	17.16
**n2**	25.80	25.94	25.87
**n3**	35.17	35.29	35.22
**n4**	32.75	32.93	32.97
**i1**		25.77	25.69
**i2**		31.11	31.04
**i3**		42.43	42.38
**a1**		14.44	14.42
**a2**		33.02	32.80
**a3**		38.04	37.62
**a4**		40.23	40.18
**a5**		30.96	30.69

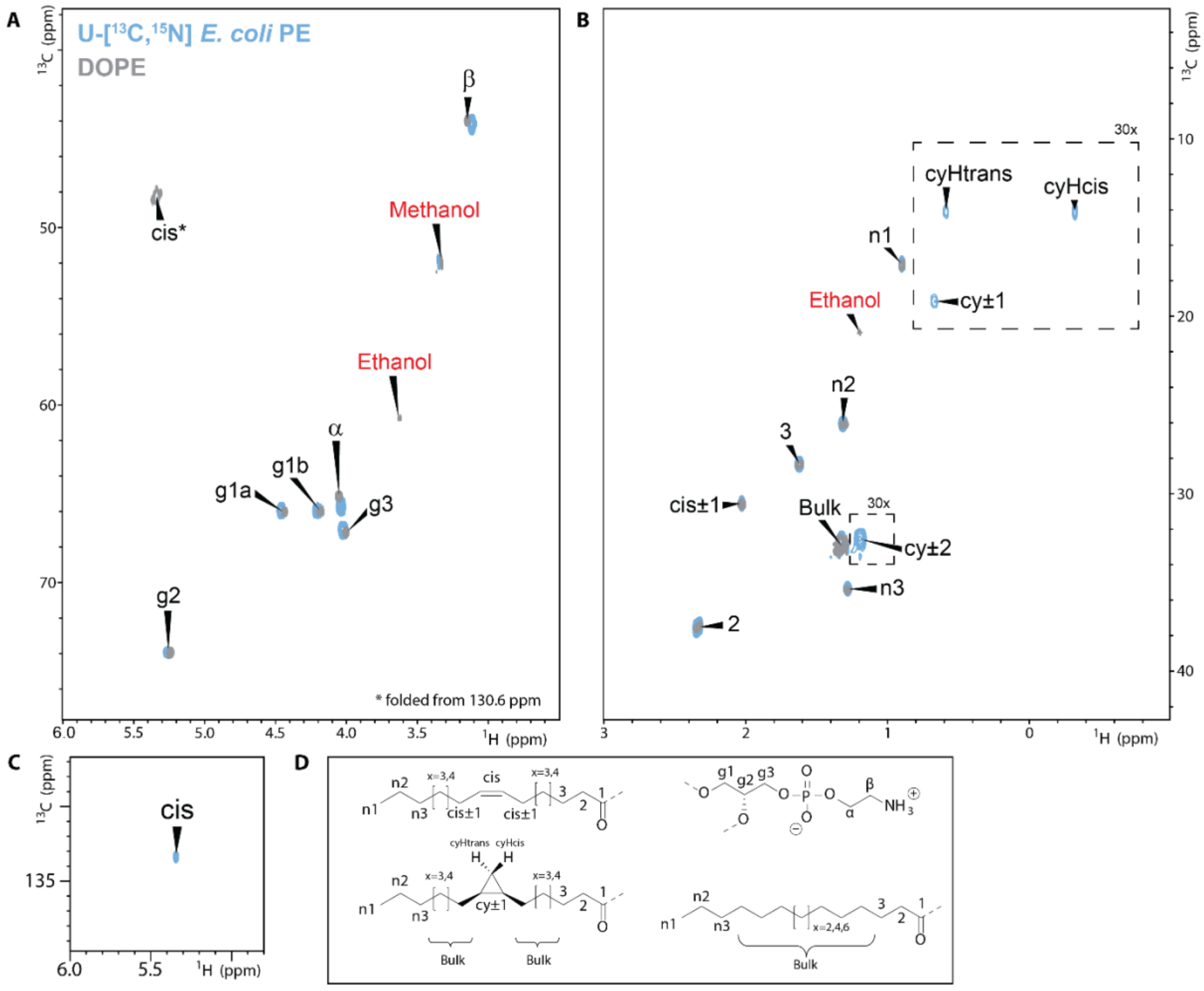

Figure S1: Solution state NMR assignments of isolated U-[^{13}C,^{15}N]-PE from *E. coli* and commercial DOPE in 2:1 $CD_3OD/CDCl_3$. A) Overlay of ^{1}H-^{13}C-(CT-)HSQC spectra of the headgroup region; B) Overlay of ^{1}H-^{13}C-(CT-)HSQC spectra of the headgroup region; C) Overlay of ^{1}H-^{13}C-(CT-)HSQC spectra of the alkene region; D) Substructures of commercial and isolated PE with assignment nomenclature.

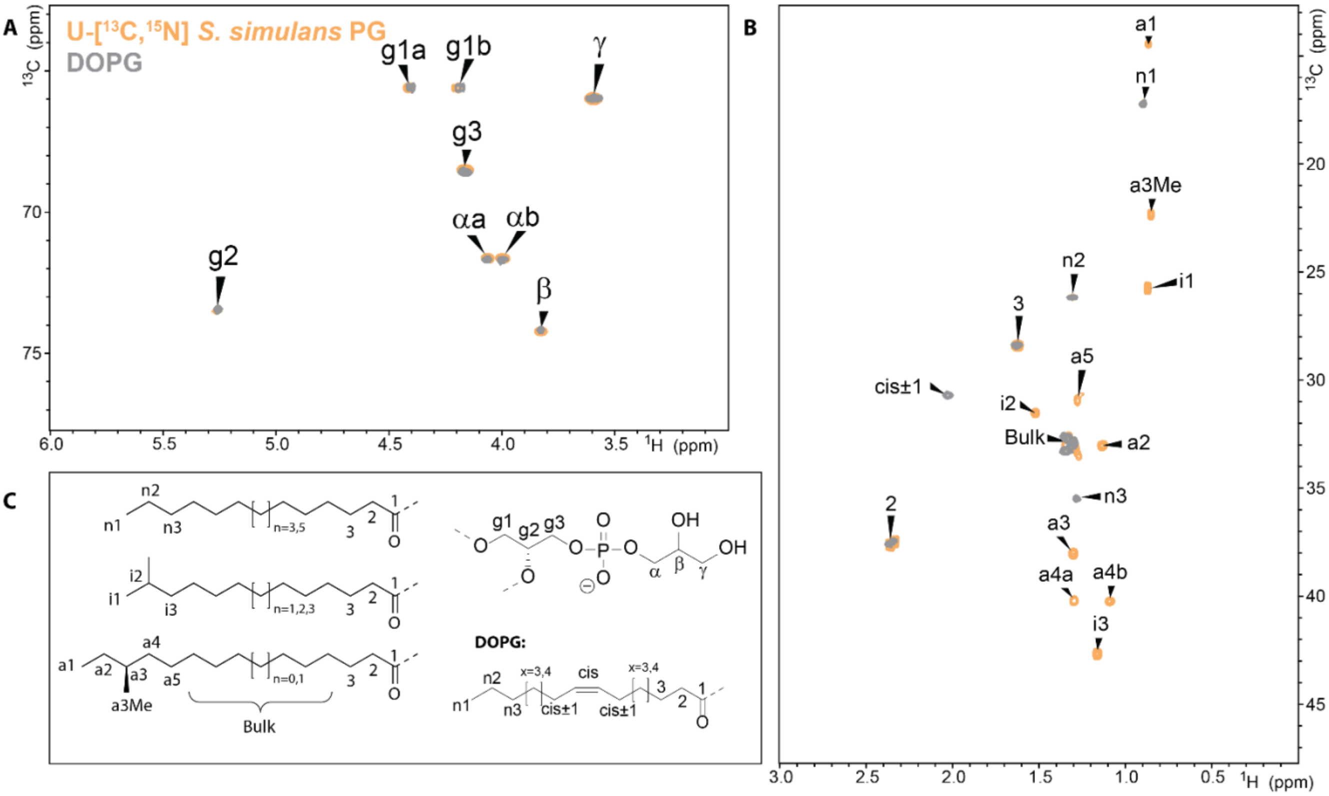

Figure S2: Solution state NMR assignments of isolated U-[^{13}C,^{15}N]-PG from *S. simulans* and commercial DOPG in 2:1 $CD_3OD/CDCl_3$. A) Overlay of ^{1}H-^{13}C-(CT-)HSQC spectra of the headgroup region; B) Overlay of ^{1}H-^{13}C-(CT-)HSQC spectra of the headgroup region; C) Substructures of commercial and isolated PG with assignment nomenclature.

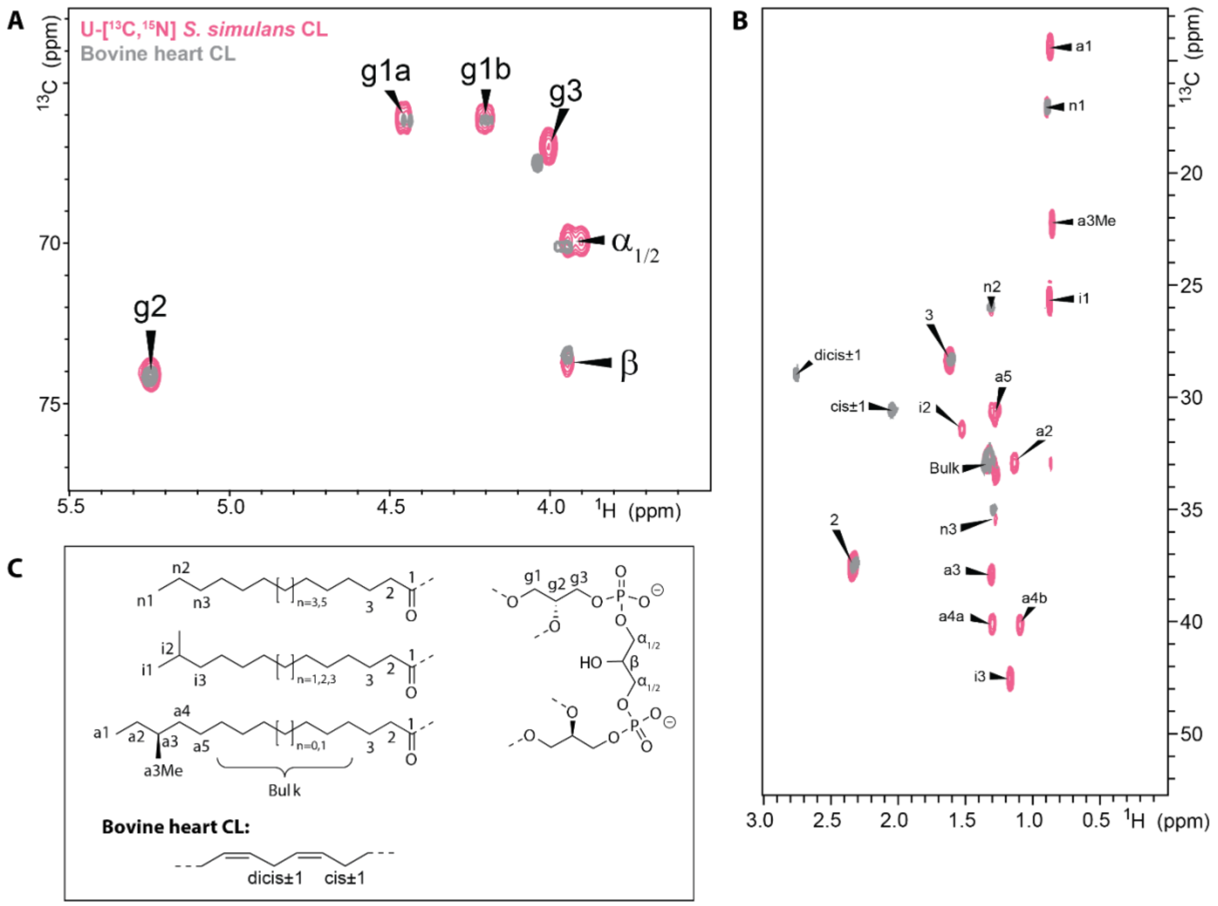

Figure S3: Solution state NMR assignments of isolated U-[$^{13}C^{15}N$]-CL from *S. simulans* and commercial bovine heart CL in 2:1 $CD_3OD/CDCl_3$. A) Overlay of ^1H-^{13}C-(CT-)HSQC spectra of the headgroup region; B) Overlay of ^1H-^{13}C-(CT-)HSQC spectra of the headgroup region; C) Substructures of commercial and isolated CL with assignment nomenclature.

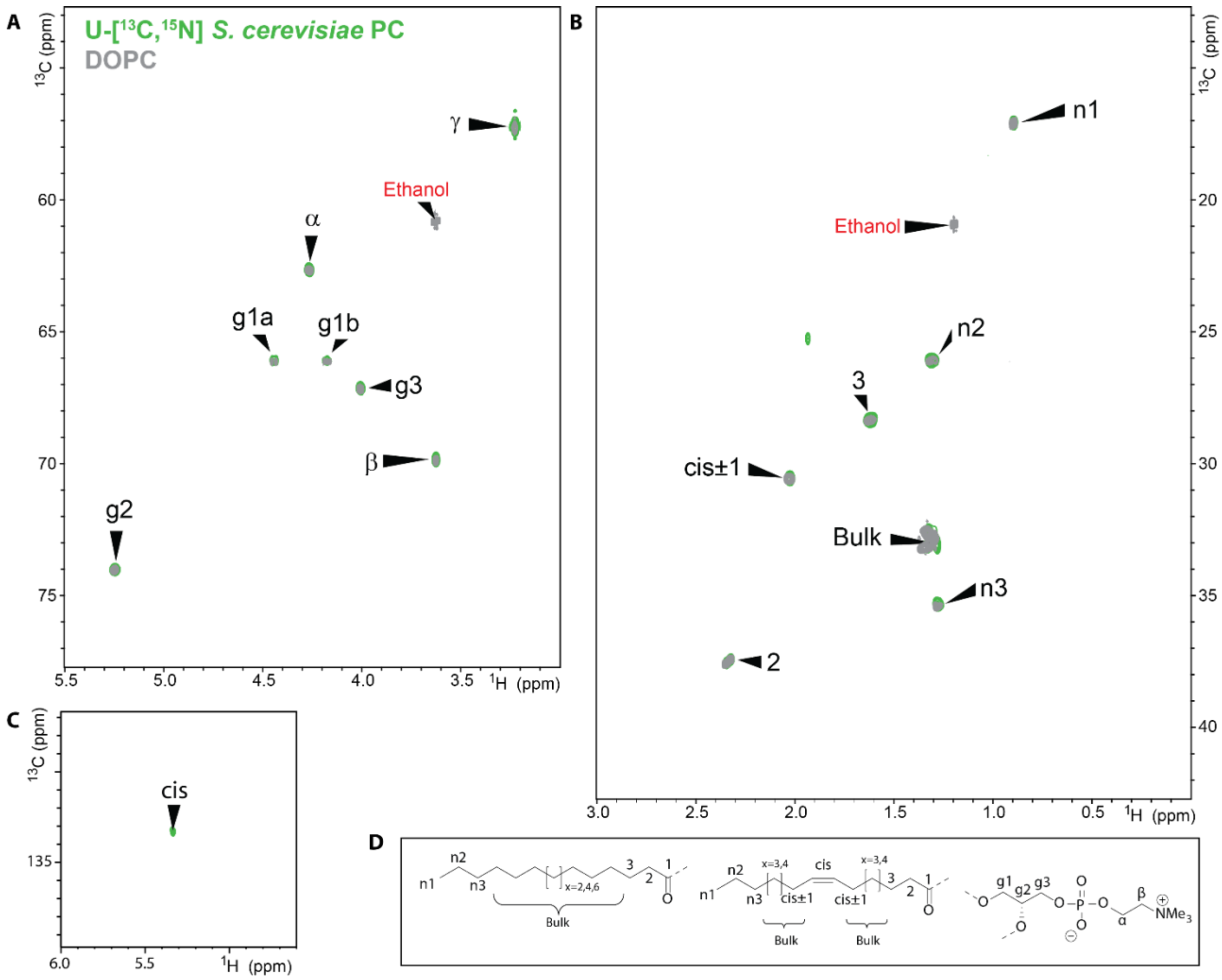

Figure S4: Solution state NMR assignments of isolated U-[$^{13}C^{15}N$]-PC from *Sa. cerevisiae* and commercial DOPC in 2:1 $CD_3OD/CDCl_3$. A) Overlay of ^1H-^{13}C-(CT-)HSQC spectra of the headgroup region; B) Overlay of ^1H-^{13}C-(CT-)HSQC spectra of the headgroup region; C) Overlay of ^1H-^{13}C-(CT-)HSQC spectra of the alkene region; D) Substructures of commercial and isolated PC with assignment nomenclature.

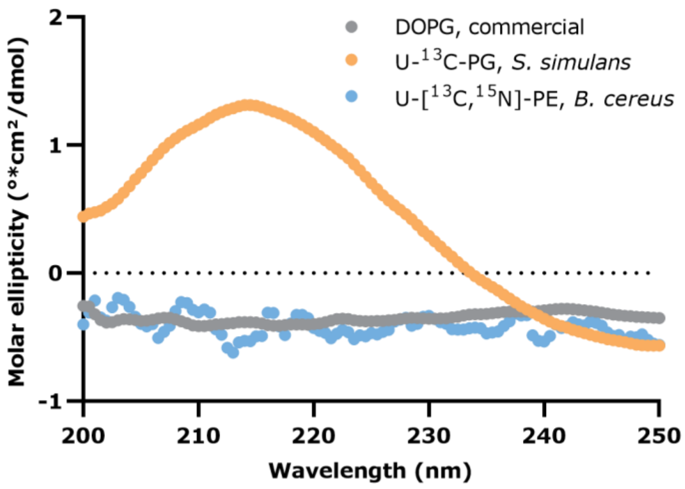

Figure S5: The headgroup of isolated PG is present in enantiomeric excess. Comparison of commercial DOPG, *U*-^{13}C-PE from *Bacillus cereus* and *U*-^{13}C-PG from *Staphylococcus simulans*. The present analysis contains PE from *B. cereus* to rule out contributions from *anteiso* fatty acids and the natural chirality of the glycerol backbone. CD spectra were recorded on a Jasco J-810-150S spectropolarimeter, using a 1 mm quartz cuvette at 20 °C. Lipids were dissolved to 1 g/L in MeOH. Obtained spectra were corrected for a MeOH blank. Spectra were averaged over five scans and represent data for which the absorbance was ≤2.0.

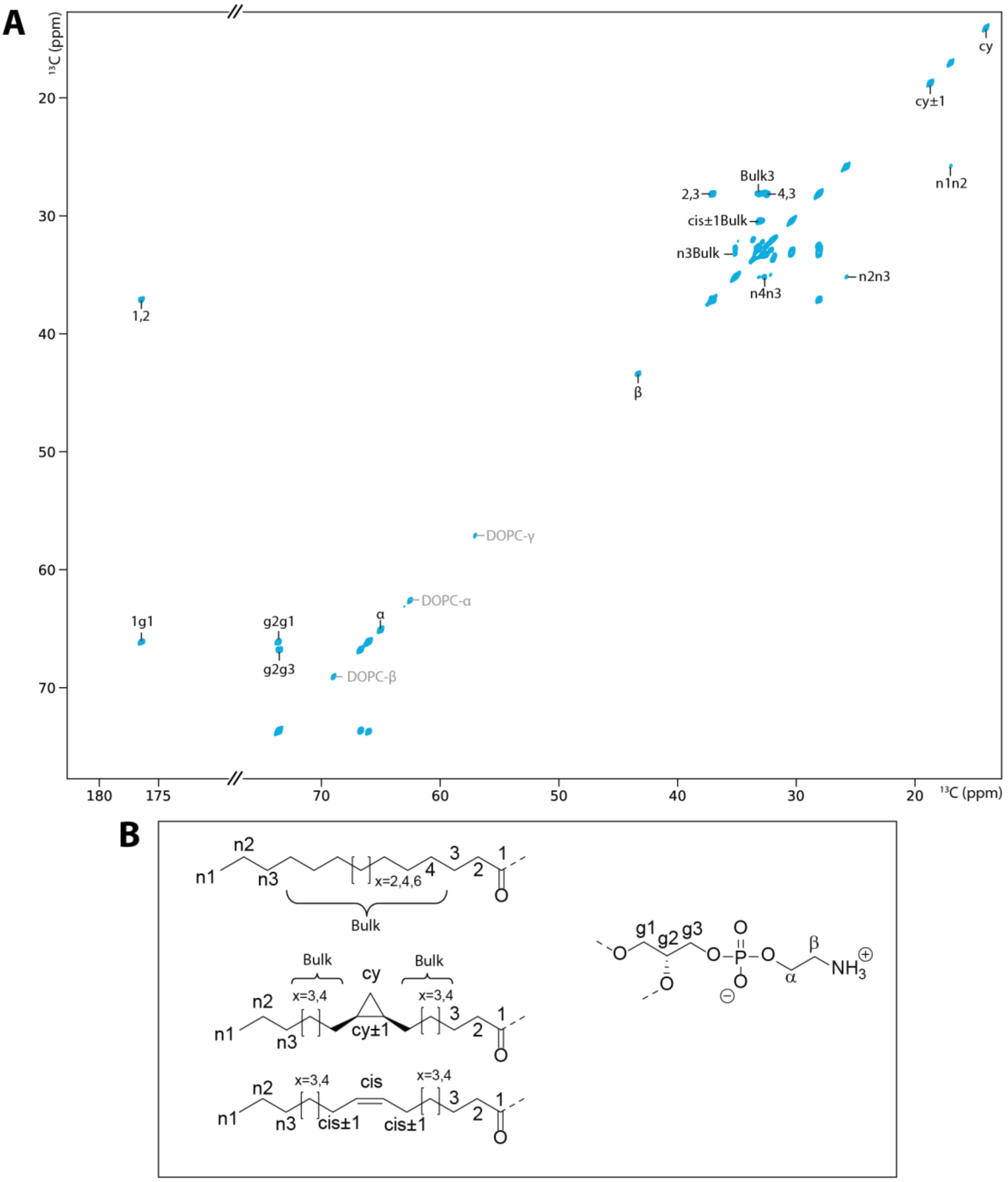

Figure S6: Solid state NMR assignments of PE from *E. coli* in DOPC membranes. A) 2D ^{13}C-^{13}C PARISxy spectrum acquired at 1200 MHz (^{1}H frequency) and 17 kHz MAS, using a mixing time of 200 ms and with a sample temperature of 275 K; B) Substructures of *E. coli* PE with assignment nomenclature.

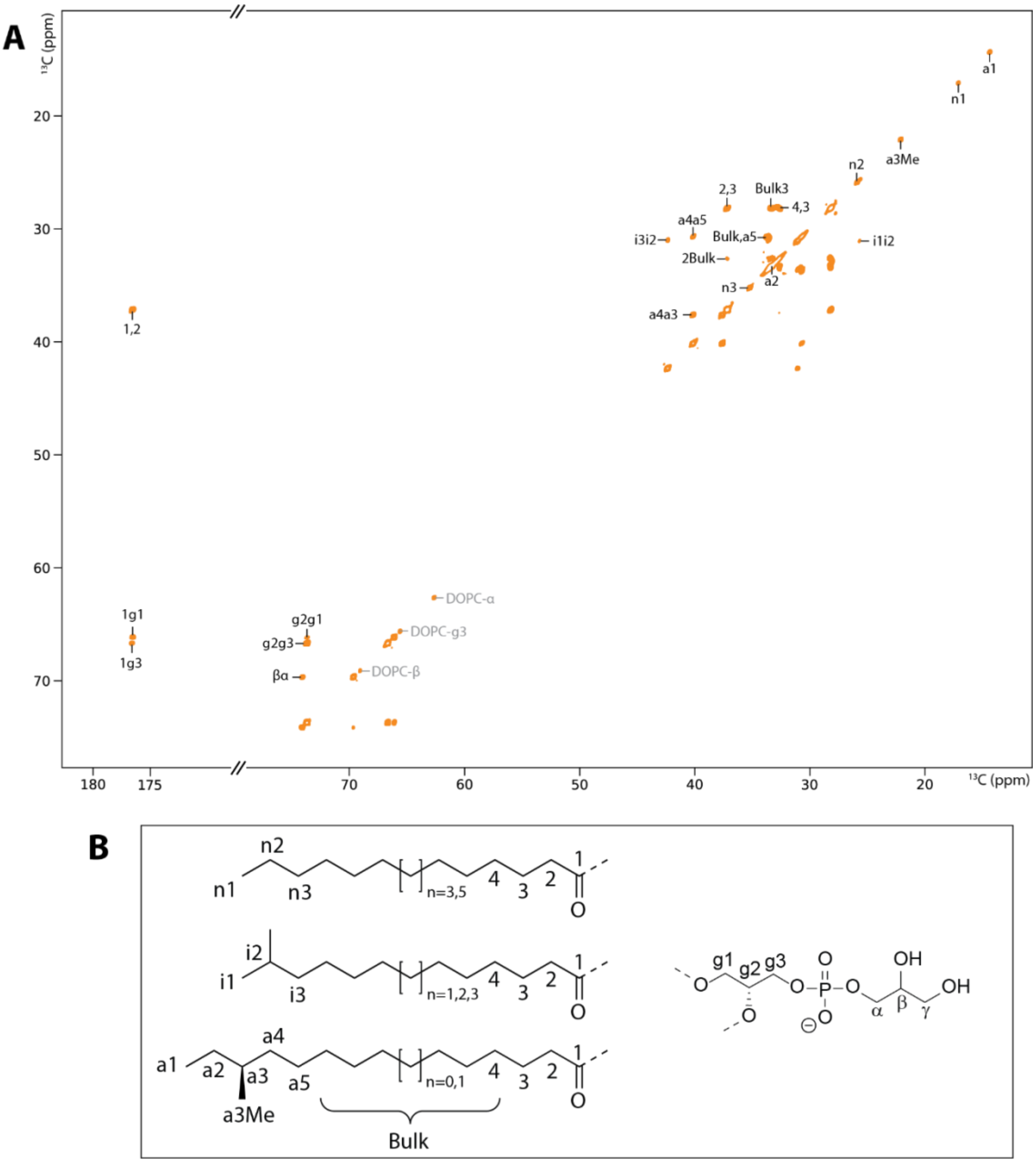

Figure S7: Solid state NMR assignments of PG from *S. simulans* in DOPC membranes. A) 2D ^{13}C-^{13}C PARISxy spectrum acquired at 1200 MHz (^{1}H frequency) and 17 kHz MAS, using a mixing time of 200 ms and with a sample temperature of 275 K; B) Substructure of *S. simulans* PG with assignment nomenclature.

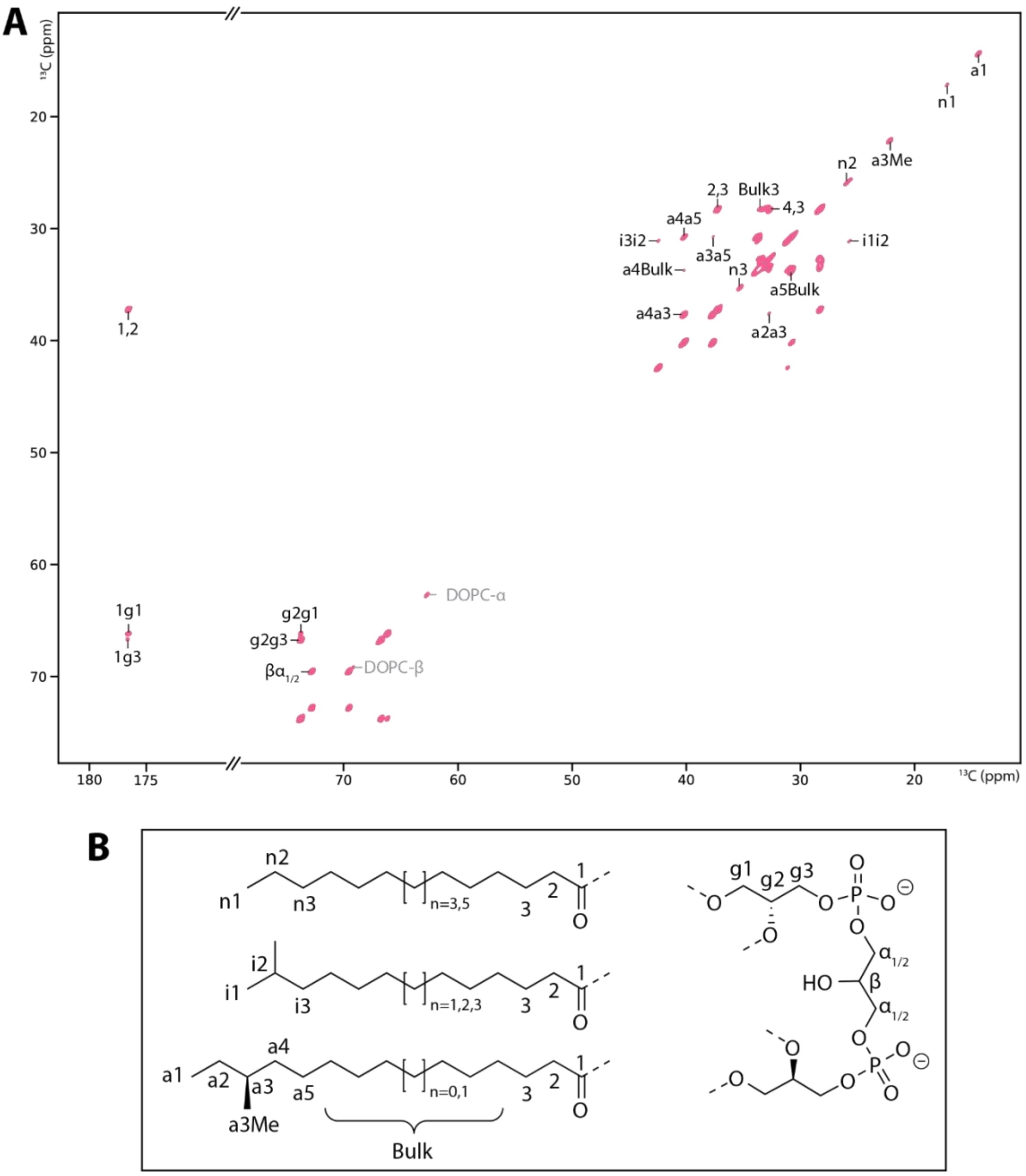

Figure S8: Solid state NMR assignments of CL from *M. flavus* in DOPC membranes. A) 2D ^{13}C-^{13}C PARISxy spectrum acquired at 1200 MHz (^{1}H frequency) and 17 kHz MAS, using a mixing time of 200 ms and with a sample temperature of 275 K; B) Substructure of *M. flavus* CL with assignment nomenclature.

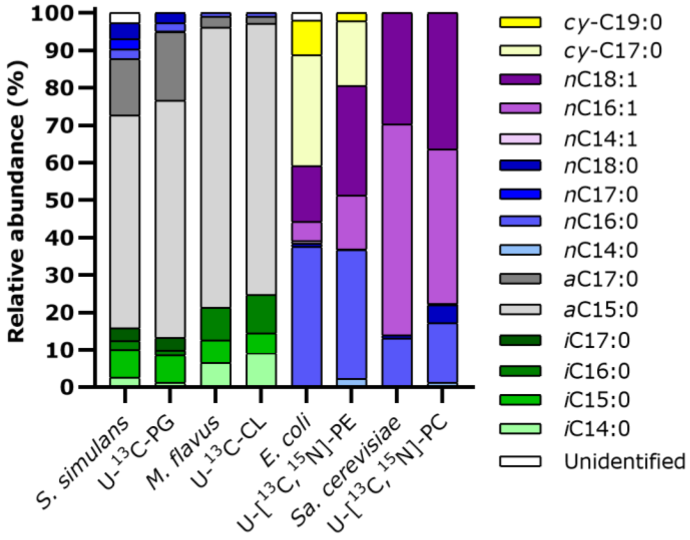

Figure S9: Relative acyl chain distributions of the isolated isotopically enriched phospholipids compared to total lipid extracts of source bacterium grown in LB or yeast in SD.

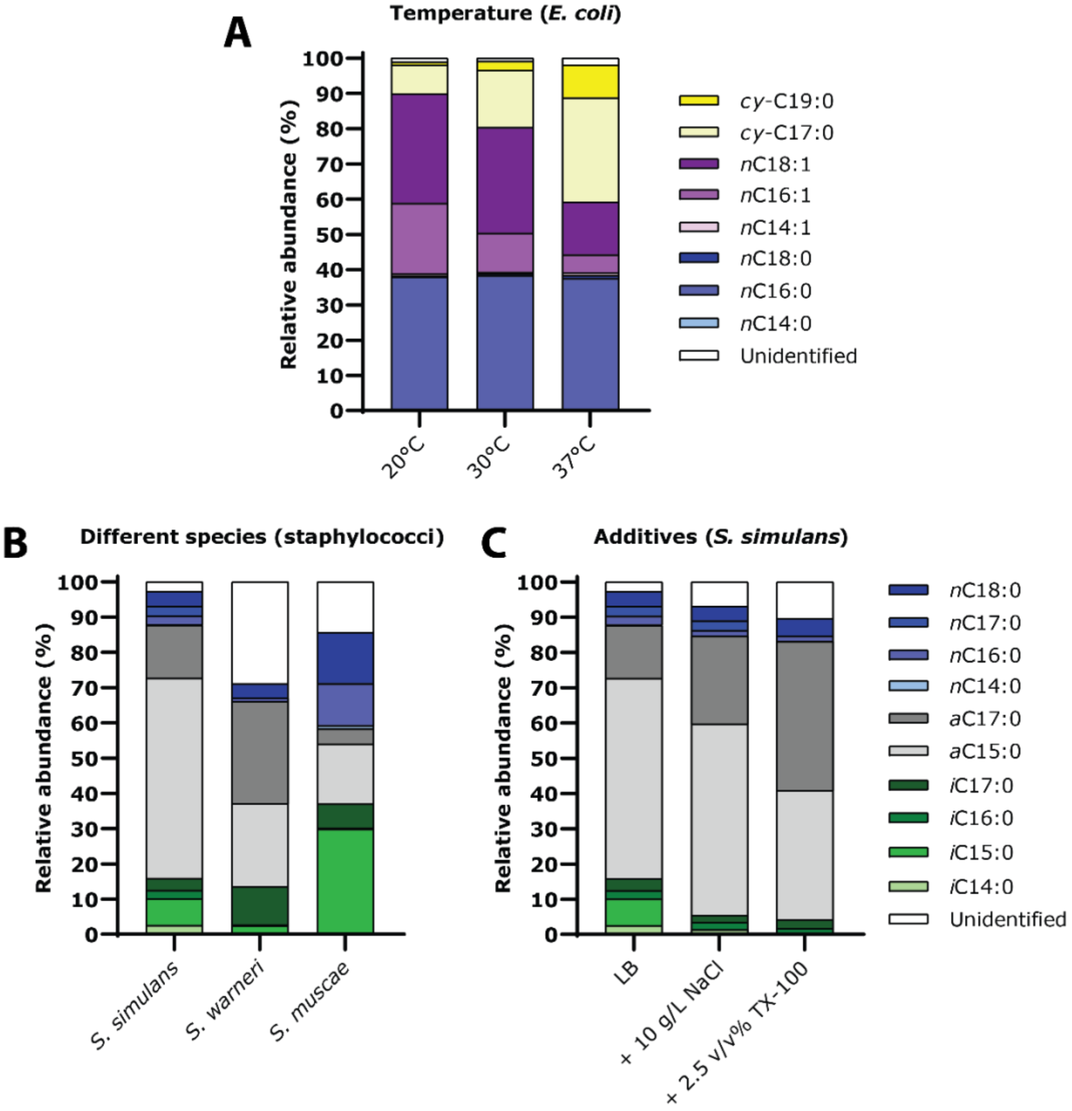

Figure S10: Relative acyl chain distributions when certain nutrients are supplied to the bacteria or when certain growth conditions are altered. Bacteria were grown in LB & 37 °C unless specified otherwise. A) Effect of cultivation temperature on *E. coli*; B) Different staphylococci yield different ratios of iso/anteiso and straight fatty acid tails. Unidentified species are longer chains than *n*C18:0, presumably *n*C20[1]; C) Effect of the addition of salt or detergent to the fatty acid distribution of *S. simulans*.

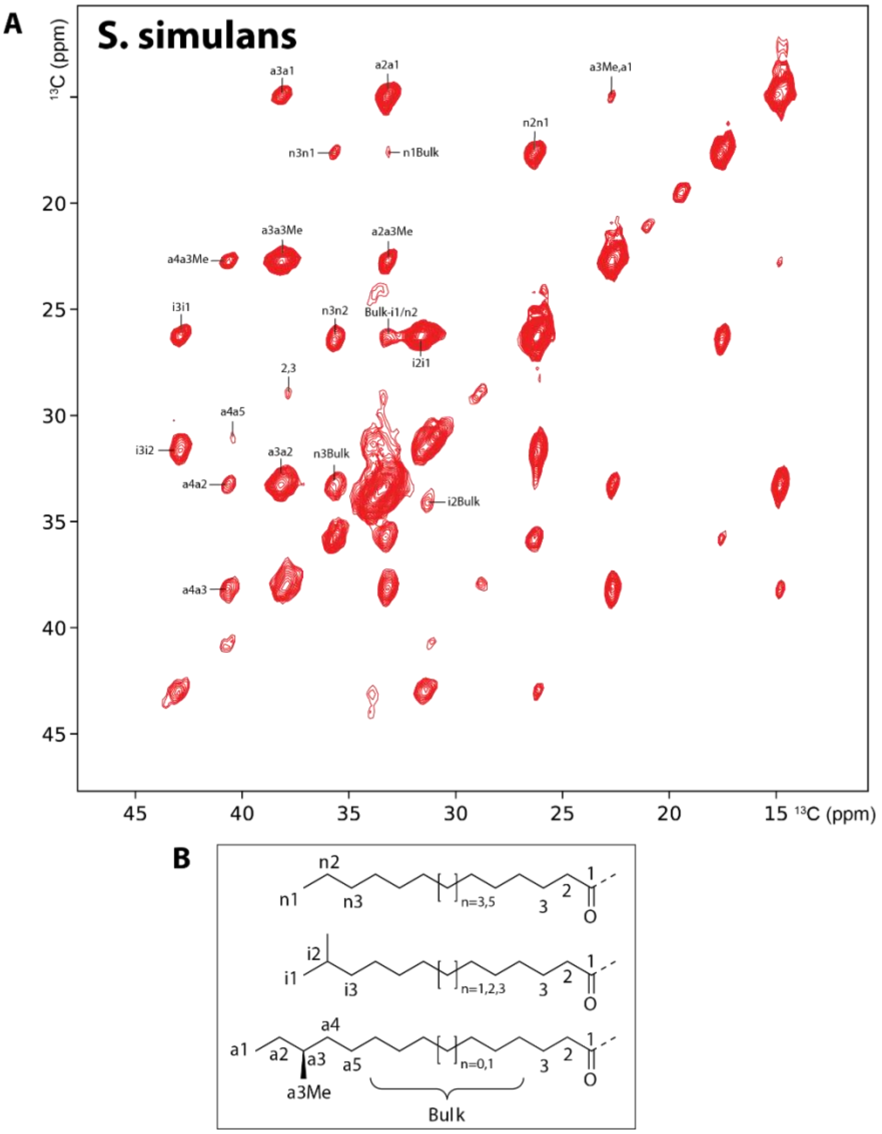

Figure S11: Correlations between mobile carbons of the lipid tails at high temperatures can readily be observed with scalar magnetization transfer. A) ^{13}C-^{13}C TOBSY spectrum of U-[^{13}C^{15}N]-lipids from *S. simulans* in DOPC membranes. Spectrum was acquired at a magnetic field of 700 MHz (^{1}H frequency) with 8 kHz MAS, sample temperature of 305 K and a CC mixing time of 6 ms; B) Substructures of *staphylococcal* lipid tails with assignment nomenclature.

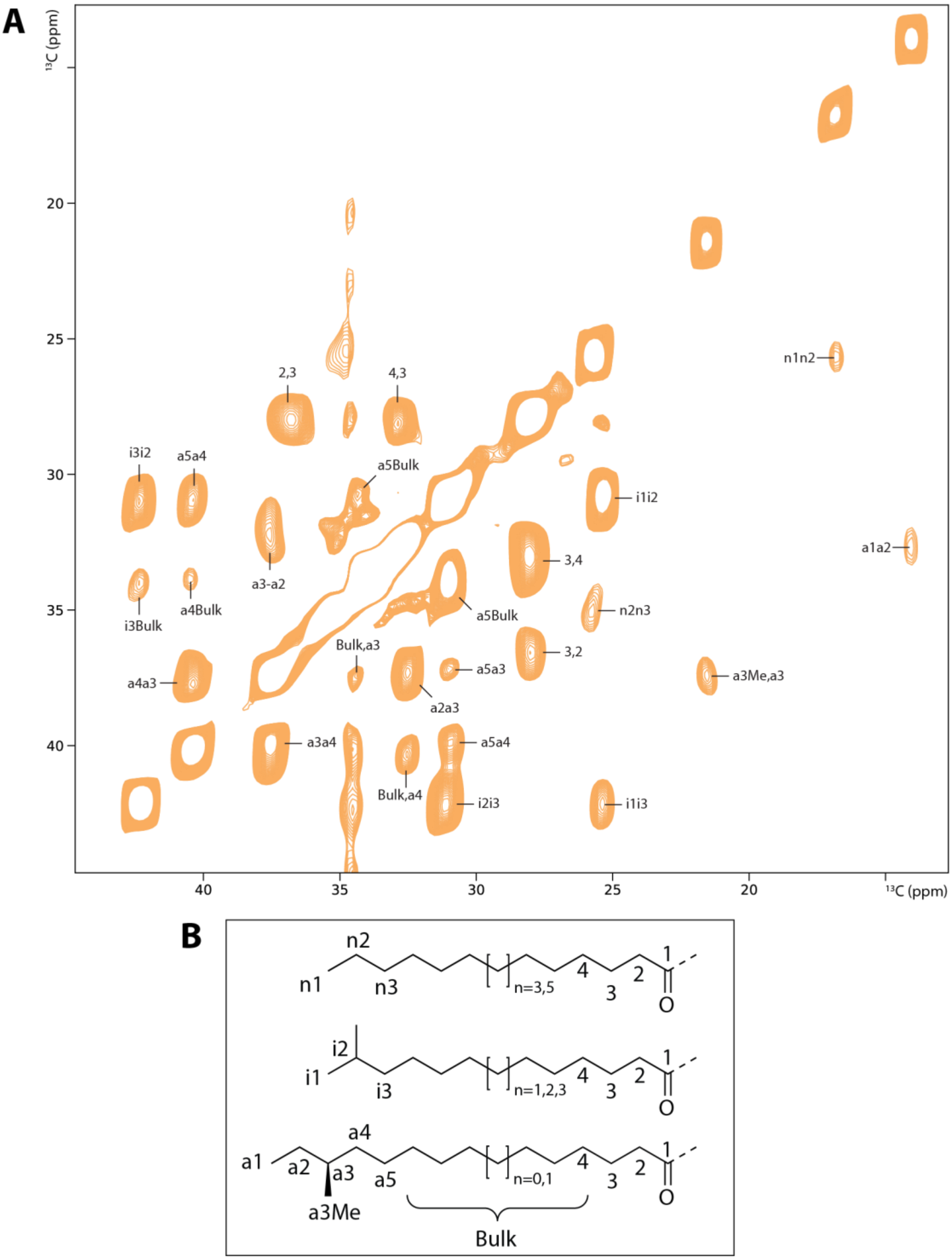

Figure S12: Correlations between carbons near the end of the lipid tail are only observed at reduced temperatures. A) 2D ^{13}C-^{13}C PARISxy spectrum acquired at 500 MHz (^{1}H frequency) and 12 kHz MAS, using a mixing time of 150 ms and with a sample temperature of 255 K; B) Substructures of *staphylococcal* lipid tails with assignment nomenclature.

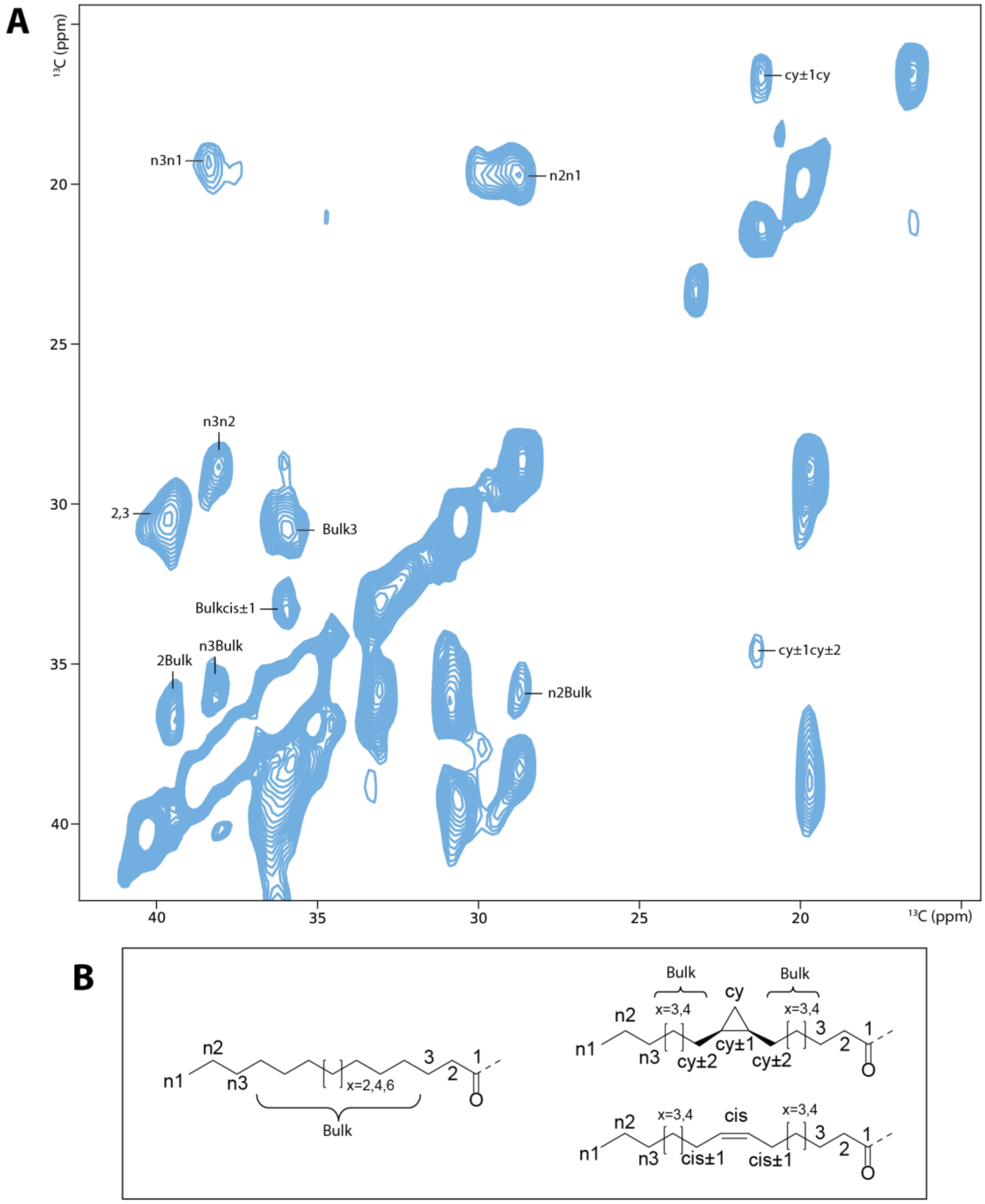

Figure S13: Correlations between carbons near the end of *E. coli* lipid tail are only observed at reduced temperatures. A) 2D ^{13}C-^{13}C PARISxy spectrum acquired at 500 MHz (^{1}H frequency) and 12 kHz MAS, using a mixing time of 150 ms and with a sample temperature of 255 K; B) Substructures of *E. coli* lipid tails with assignment nomenclature.

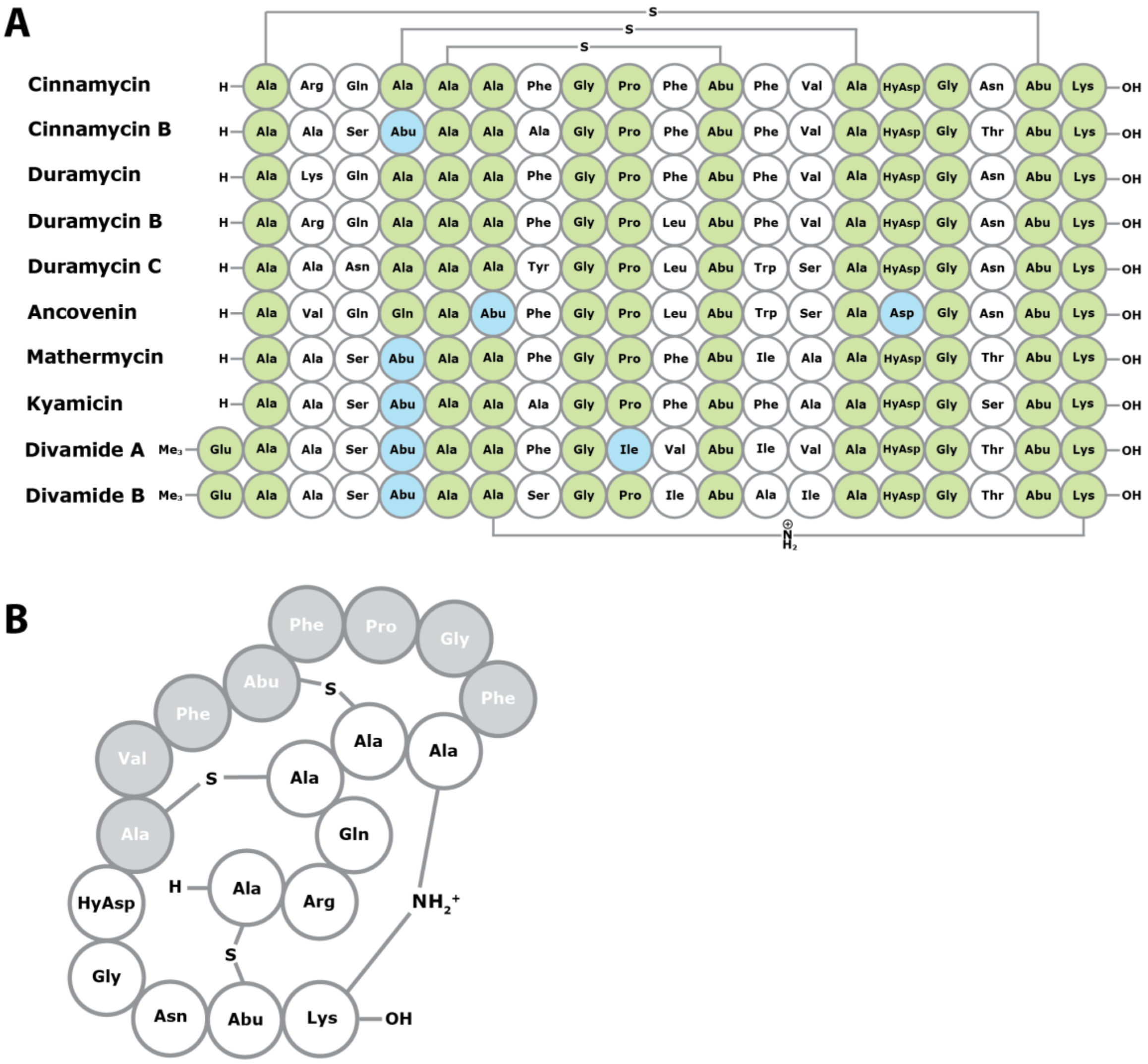

Figure S14: Overview of cinnamycins. Abu: L-2-aminobutyric acid; HyAsp: *erythro*-3-hydroxy-aspartic acid. A) Alignment of all known cinnamycin-class lantibiotics. Homologies are indicated with the colors green and blue. Note that ancovenin, contrary to the others, does not have a lysinoalanine-bridge. B) Schematic representation of cinnamycin. The presumed hydrophobic membrane-interacting stretch is depicted in grey.

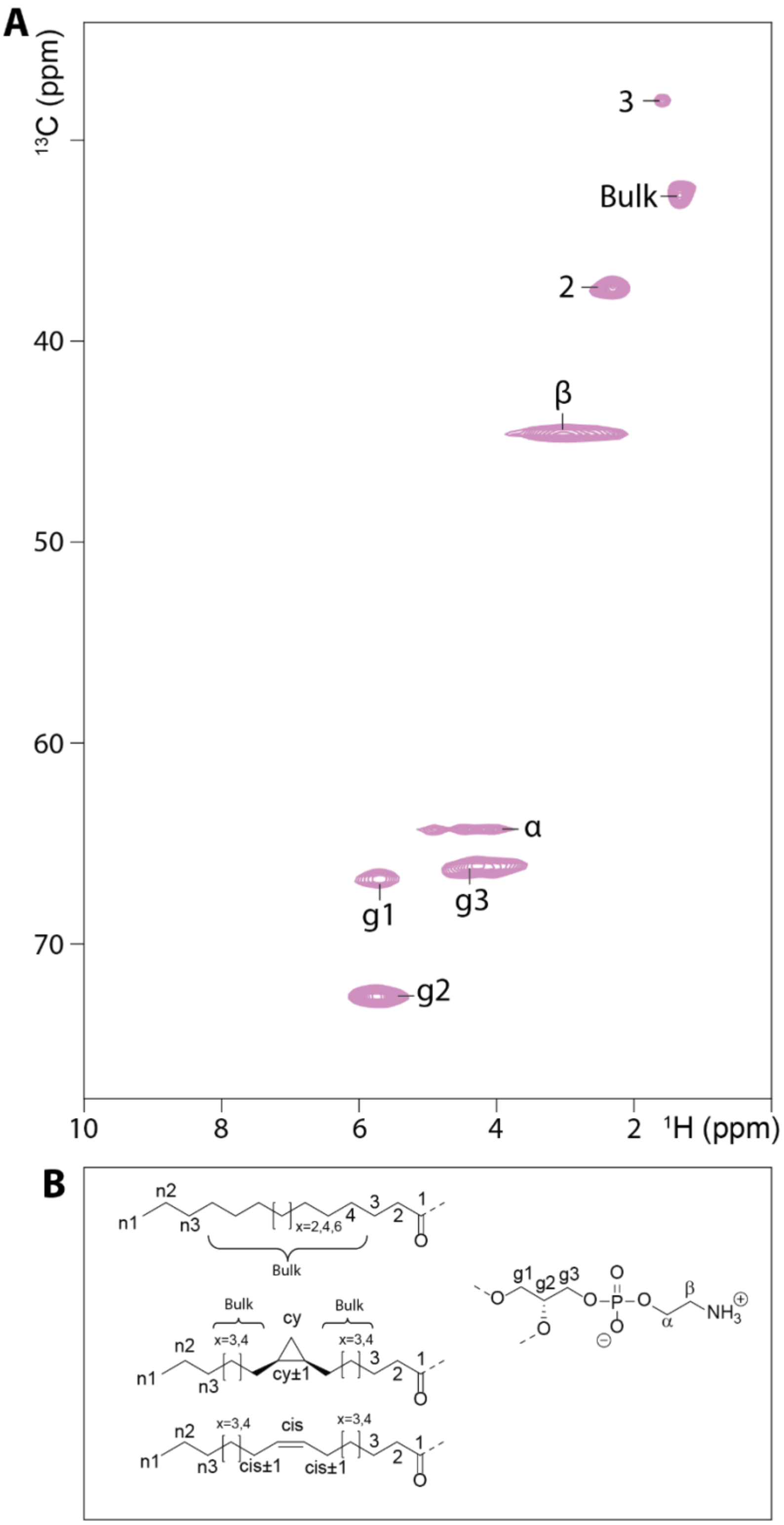

Figure S15: ^{1}H detected ^{13}C-^{1}H spectra of cinnamycin-bound *U*-[^{13}C,^{15}N]-PE from *E. coli* in DOPC membranes A) 2D ^{13}C-^{1}H spectrum acquired at 700 MHz (^{1}H frequency) and 60 kHz MAS, using cross-polarization for magnetization transfer and a sample temperature of 305 K; B) Substructures of *U*-[^{13}C,^{15}N]-PE from *E. coli* with assignment nomenclature.

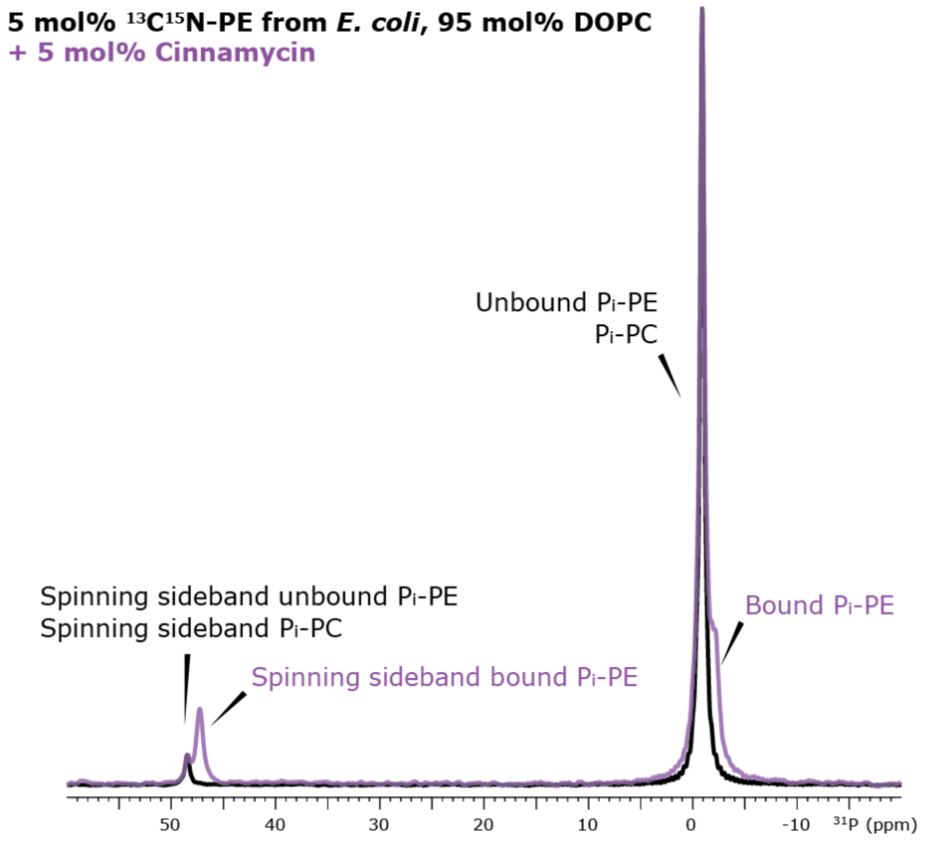

Figure S16: Overlay of 1D ^{31}P solid state NMR spectra of 5 mol% U-[^{13}C,^{15}N]-PE from E. coli in DOPC in the absence (black) and presence (purple) of 5 mol% cinnamycin. Spectra were recorded at a magnetic field of 500 MHz (^{1}H frequency), 10 kHz MAS and at a sample temperature of 275 K.

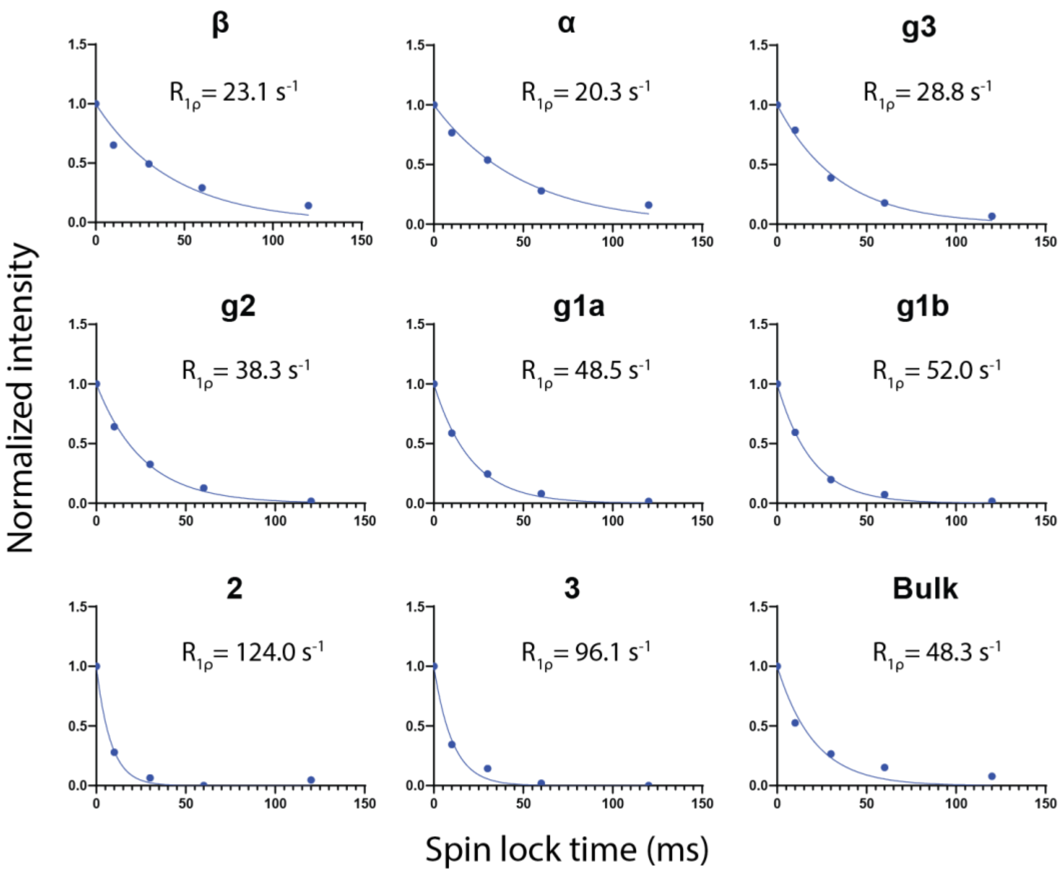

Figure S17: Overview of ^{13}C $R_{1\rho}$ data of cinnamycin-bound U-[^{13}C,^{15}N]-PE from *E. coli*. 2D ^{1}H-detected (H)CH spectra were recorded at 60 kHz MAS, a magnetic field of 700 MHz (^{1}H frequency) and a sample temperature of 305 K. An 18 kHz spin-lock pulse of 0, 10, 30, 60 or 120 ms was applied on ^{13}C directly after the first CP. Peaks were integrated, normalized to the experiment without spin-lock and fit with a single exponential decay.

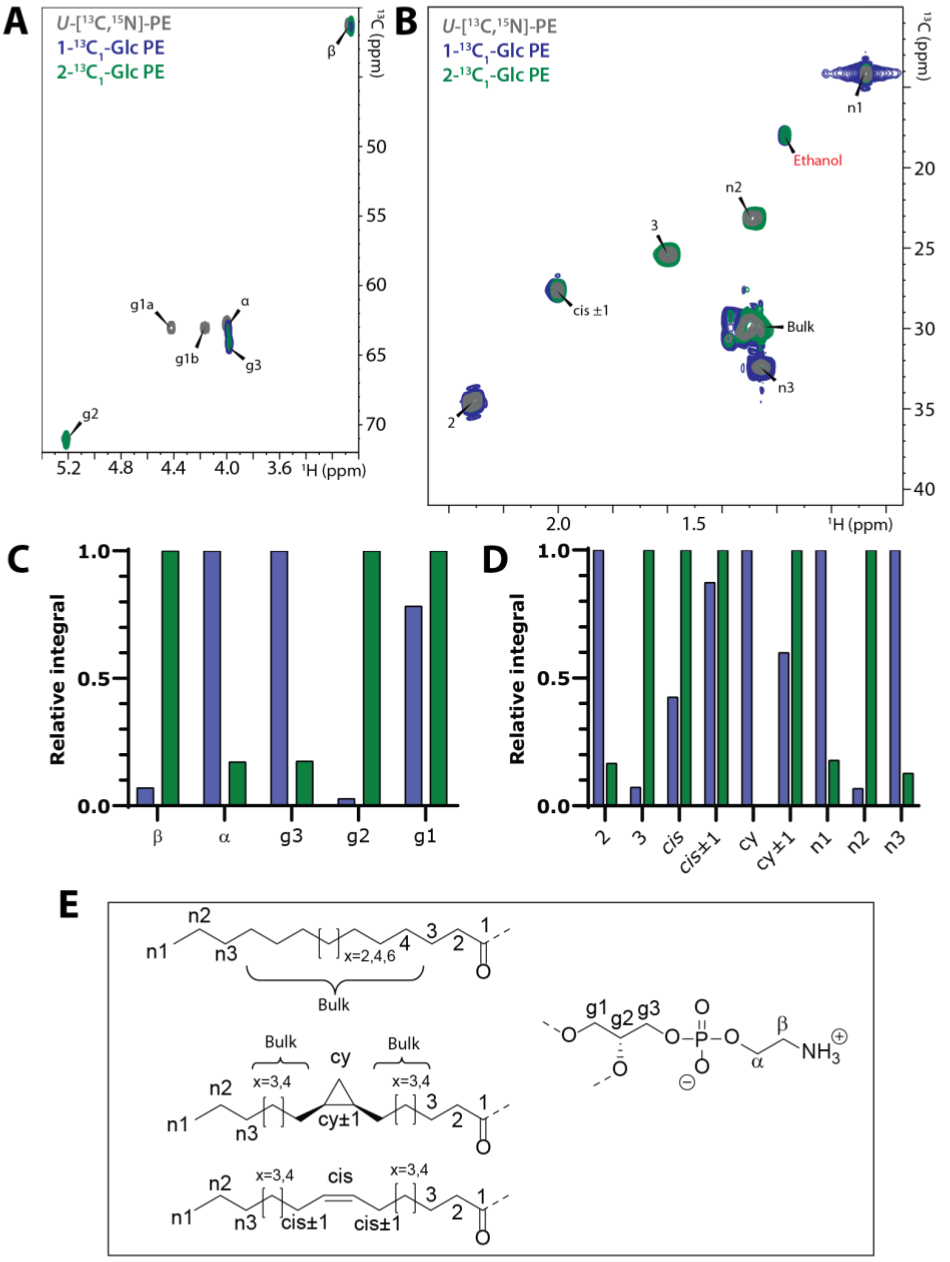

Figure S18: Selective ^{13}C labelling of PE. PE was isolated from E. coli grown in M9 minimal medium supplemented with 2 g/L 1-$^{13}C_1$-D-Glucose, 2-$^{13}C_1$-D-Glucose or *U*-^{13}C-D-Glucose A) and B) Overlay of ^{1}H-^{13}C-CT-HSQC spectra displaying the headgroup and tail region respectively; C) and D) Relative integrals 1- and 2-$^{13}C_1$-D-Glucose labeled PE. Highest peak integral of the two labeling schemes was arbitrarily set to 1. A ^{1}H 1D was used to correct for varying lipid concentrations in the samples; E) Substructures of *E. coli* PE with assignment nomenclature.

Supporting references

[1] W. M. O'Leary, S. G. Wilkinson, in *Microbial Lipids, Vol. 1* (Eds.: C. Ratledge, S. G. Wilkinson), Academic Press, **1988**, pp. 117-201.
